# Towards Cytotoxic Derivatives of Cafestol

**DOI:** 10.3390/molecules30112291

**Published:** 2025-05-23

**Authors:** Niels V. Heise, Marie Kozubek, Sophie Hoenke, Senta Ludwig, Hans-Peter Deigner, Ahmed Al-Harrasi, René Csuk

**Affiliations:** 1 Organic Chemistry, Martin-Luther-University Halle-Wittenberg, Kurt-Mothes Str. 2, 06120 Halle (Saale), Germany; 2 Institute of Precision Medicine, Furtwangen University, Jakob-Kienzle-Str. 17, 78054 Villingen-Schwenningen, Germany; 3 Natural and Medical Sciences Research Center, University of Nizwa, Birkat Al-Mauz, P.O. Box 33, Nizwa 616, Oman

**Keywords:** cafestol, kahweol, cytotoxicity

## Abstract

This study focuses on the extraction, characterization, and biological evaluation of diterpenes from green coffee beans, specifically, cafestol and kahweol. These compounds, known for their potential health benefits, were isolated via optimized extraction and saponification processes. Separation was achieved using silver nitrate-impregnated silica gel, and structural elucidation was performed through advanced 1D and 2D NMR techniques, including HSQC, HMBC, and (IN)ADEQUATE. Due to kahweol’s instability, the research prioritized cafestol for the synthesis of rhodamine B conjugates. Initial ester-linked conjugates proved unstable, prompting the development of more robust derivatives through amide linkage strategies and further functionalization via acetylation and oxidation reactions. Some oxidation methods led to furan ring cleavage, impacting structural integrity. Selected compounds were tested for cytotoxicity using SRB assays on human tumor cell lines (MCF7, A2780) and non-malignant fibroblasts (NIH 3T3). While the parent diterpenes and many derivatives showed minimal activity, several cafestol–rhodamine B conjugates demonstrated notable cytotoxic effects. Compound **6**, in particular, exhibited selective activity against cancer cells with reduced toxicity toward non-malignant cells.

## 1. Introduction

Coffee is one of the most popular beverages in the world. The coffee plant originates from the Kaffa region in southwestern Ethiopia and was mentioned as early as 900 AD. While at that time, the leaves and dried cherries—similar to tea—were infused in hot water and then drunk, nowadays, the raw, dry seeds are roasted, finely ground, and boiled in water [1,2,3,4].

**Figure 1:**
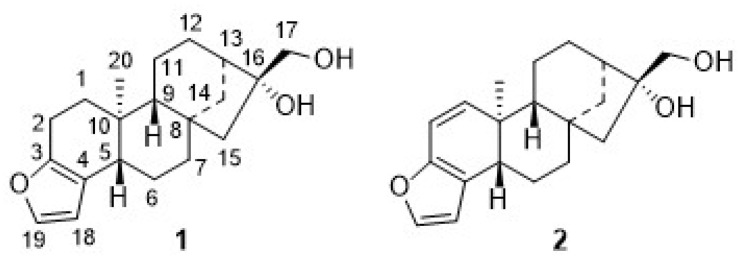
Structures of cafestol (**1**) and kahweol (**2**) and their numbering schemes.

Coffee contains more than 1000 different substances, and physiological effects are attributed to many of them. The most recent development is the so-called “green coffee”: this is unroasted coffee, freed of pulp and seed husk. Enjoying this “green coffee” was claimed to reduce weight, but also to reduce the risk of diabetes and even slow down the aging process. However, there are also indications that the regular use of green coffee is able to reduce liver and colon cancer and lower the risk of breast and prostate cancer. This is probably due to the presence of chlorogenic acids. Green coffee is also rich in heat-, light-, and acid-labile diterpenes, such as cafestol (**1**, Figure 1) and kahweol (**2**) [5,6,7,8,9]. The latter compound was first isolated in 1932 by Bengis and Anderson [10,11], while cafestol was described by Slotta and Neisser in 1938 [11,12]; the structures of both compounds were revealed by Djerassi [13,14,15,16,17] and Kaufmann et al. [18] three decades later. Both of these *ent*-kaurane diterpenes were reported to be slightly cytotoxic and show some antitumor activity. There are only a few reports about their bioactivity, and even fewer about the cytotoxic properties of the derivatives thereof [3,19,20,21,22].

Recently, we have shown that many rhodamine B conjugates derived from pentacyclic triterpenoic acids as well as from diterpenes hold significant cytotoxic effects for a variety of different tumor cell lines [23,24,25,26,27,28,29,30,31]. High cytotoxic activity was also observed in 3D spheroids [29]. Several of these conjugates were even active in nanomolar concentrations, while significantly less cytotoxic for non-malignant cells. Their mode of action was that of a mitocan, and ongoing research showed some of them to shut down mitochondrial ATP synthesis [29].

## 2. Results

Extraction of ground green coffee beans afforded the so-called coffee oil, whose saponification left an unsaponifiable fraction, mainly composed of diterpenes. Thereby, cafestol (**1**) and kahweol (**2**) can be found in fractions originating from *Coffea canephora* (also known as robusta) or *Coffea arabica* (also known as arabica), while 16-*O*-methyl-cafestol is a molecular marker for the former species [3].

Many procedures have been published for the extraction of diterpenes **1** and **2**; these procedures differ in solvents, temperature, way of extraction (batch or continuous), and the sequence of different purification and extraction steps [2,32]. Classical chromatographic separation of **1** and **2** is tedious, often not reliable, and scaling up is always difficult due to the small differences in structure between **1** and **2** [33,34,35].

**Scheme 1:**
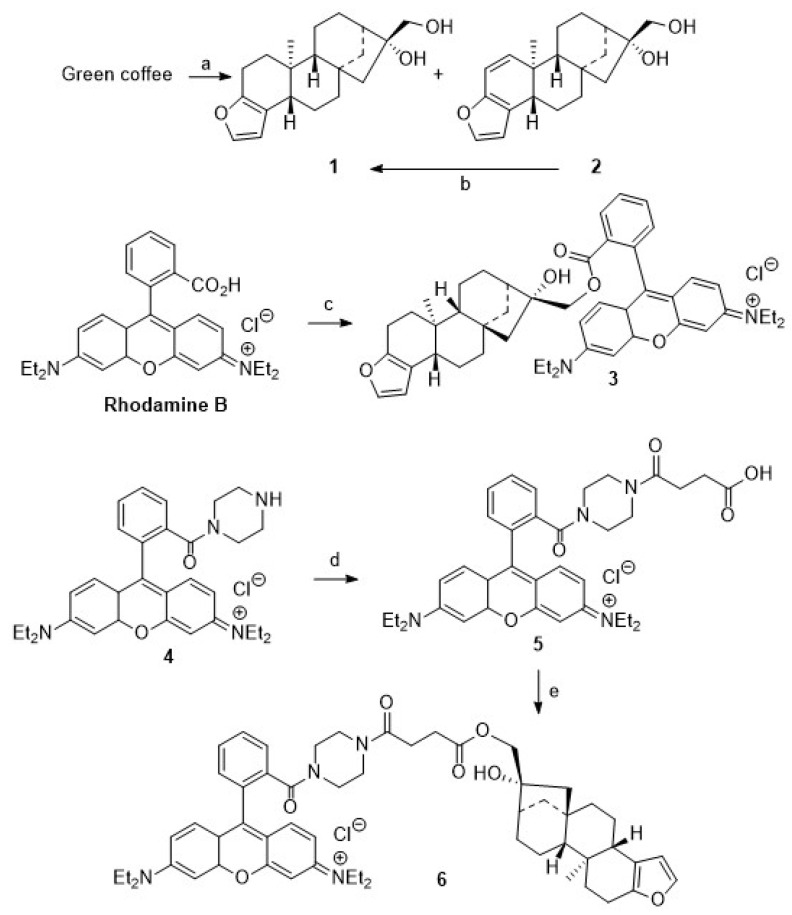
Reactions and conditions: (a) MTBE extraction, 12 h, 95 °C; KOH (10%) in EtOH, 95 °C, 2 h; (b) Pd/CaCO_3_ (Pb 5%), MeOH, H_2_ (3 bar), 4 h, 98%; (c) DCM, DMAP, TEA, rhodamine B, DCC, 20 °C, 1 d, 57%; (d) DCM, TEA, MAP, succinic anhydride, 20 °C, 1 d, 75%; (e) DCM, EDC, DIPEA, DMAP, 20 °C, 3 h, 42%.

Significant amounts of **1** and **2** (Scheme 1) were obtained when either the beans were frozen with liquid nitrogen, crushed in a knife mill, and the powder was extracted with MTBE at 95 °C for 12 h in a Soxhlet apparatus or, as an alternative, spent coffee grounds were used [36,37]. The solvent was removed, and the coffee oil was saponified at 95 °C for 2 h with 10% ethanolic potassium hydroxide solution to yield a crude mixture of **1** and **2**. Earlier reports on their chromatographic separation proved unreliable and failed completely upon attempts at scaling up [33,34,35].

**Figure 2:**
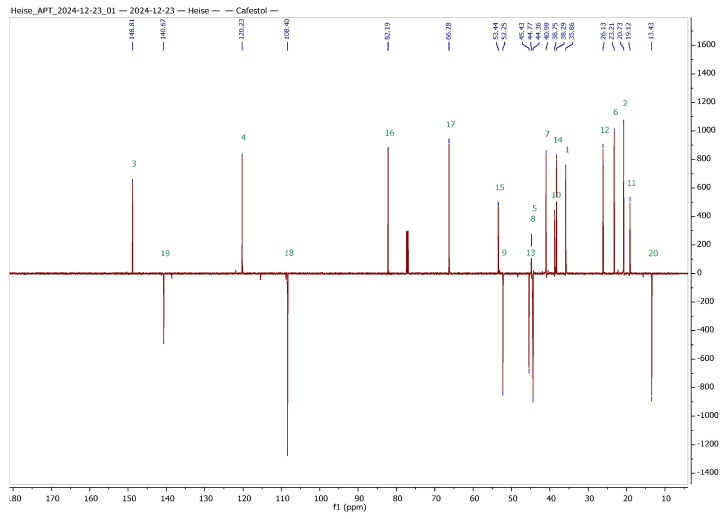

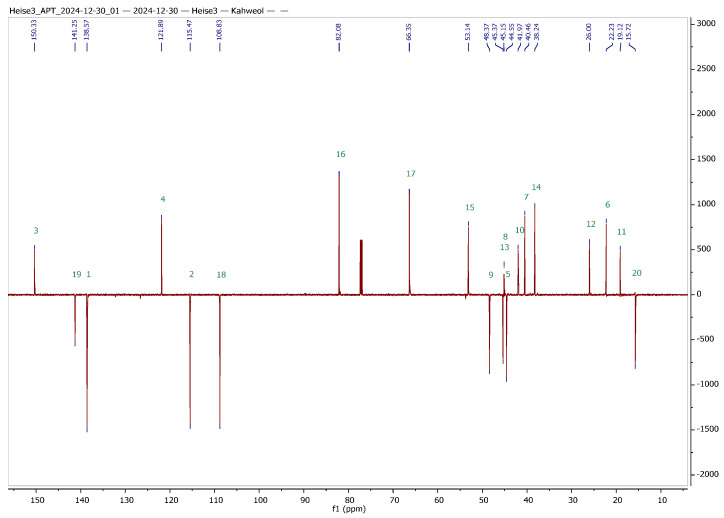
^13^C APT NMR spectra of cafestol (**top**) and kahweol (**bottom**) with complete assignment of all signals (CDCl_3_ solvent, 600 MHz, 27 °C); green numbers correspond to the carbons of the skeleton of cafestol and kahweol, respectively.

Silver nitrate-impregnated supports, however, have been used extensively for the separation of structurally related olefins, but also for the preparative separation of sesquiterpene alcohols [38,39,40,41]. Hence, we decided to try this method both for the analytical as well as for the preparative separation of **1** and **2**. The separations worked nicely on freshly prepared, self-made AgNO_3_-impregnated silica gel, and **1** and **2** were obtained in analytically pure form. To facilitate the interpretation of NMR spectra of the hybrids to be synthesized, a set of 1D and 2D NMR experiments was performed, and a complete assignment of all signals was achieved. The results from these experiments are summarized in Table 1 and Figure 2.

In order to clearly assign all signals in the ^1^H and ^13^C NMR spectra of the two terpenes (this is also a prerequisite for the clear structural clarification of the then synthesized derivatives), corresponding 2D NMR spectra were recorded. A complete assignment can already be derived from the evaluation of the gHSQC (heteronuclear single quantum coherence or heteronuclear single quantum correlation) and HMBC (heteronuclear multiple bond correlation) spectra. Independent confirmation was provided by recording INADEQUATE and ADEQUATE spectra and HSQCADTOXY spectra. The latter were recorded for comparison, as they offer the advantage of filtering out specific proton signals, and hence, TOXY-NMR spectra (total correlation spectroscopy with X-filtering) can reduce spectral congestion, making it easier to analyze complex signals.

ADEQUATE (adequate double quantum spectroscopy) and INADEQUATE (incredible natural abundance double quantum transfer experiment) are both two-dimensional NMR techniques for the determination of direct ^13^C–^13^C bonds in organic molecules. However, ADEQUATE has some significant advantages over INADEQUATE.

The former experiment usually holds higher sensitivity since ADEQUATE utilizes ^1^H-enabled detection, while INADEQUATE relies only on ^13^C signals. Since protons have a much higher natural abundance and sensitivity than ^13^C, this significantly improves the signal intensity.

Furthermore, since ADEQUATE is detected via protons, the signal-to-noise ratio (SNR) is significantly better than that of INADEQUATE. The higher sensitivity means that ADEQUATE provides meaningful spectra more quickly, whereas INADEQUATE often requires extremely long measurement times. ADEQUATE can be performed with smaller sample quantities as ^1^H detection provides much better sensitivity. Although HMBC and HSQC experiments provided essential structural information, both INADEQUATE and 1,1-ADEQUATE experiments were performed to directly observe ^13^C connectivity and validate the structural assignments. We are well aware that either INADEQUATE or ADEQUATE would likely have sufficed, but we used the opportunity to measure both due to extended instrument availability during institutional holidays. Notably, the INADEQUATE experiment required approximately five days of measurement time, while the ADEQUATE experiment took only two days—thus reflecting their differing sensitivities. The dual use of INADEQUATE and ADEQUATE allowed a comparison of results. It is noteworthy to mention that an HSQC or an HMBC NMR spectrum can be recorded within minutes to several hours. Since the HMBC spectrum holds lower sensitivity compared to that of HSQC, a prolonged accumulation time has to be considered. Representative NMR spectra are depicted in the Supplementary Materials.

On prolonged standing in solution, however, **2** proved to be unstable, and, as a consequence, we decided to abstain from synthesizing kahweol–rhodamine B hybrids. Resulting from this decision, we attempted to maximize the yield of **1** from the extraction process by in situ hydrogenation of **2** to yield **1**. Recently, this reaction has been investigated by Lima et al. [42,43], but better conditions and higher yields were obtained by applying a Pd/CaCO_3_ catalyst (poisoned with 5% Pb). Our results parallel previous results, as previously patented by Bertholet in 1986 [43].

For the synthesis of cafestol–rhodamine B conjugates (Scheme 1), rhodamine B was coupled with **1** using Steglich conditions, and **3** was obtained in 57% isolated yield. This compound, however, deteriorated easily, and its stability to light or even slightly acidic conditions was low due to a proven lability (as checked by TLC) of the ester bond between the rhodamine B moiety and the diterpene scaffold. To access more stable compounds, rhodamine B was converted into known piperazinyl-amide **4**. An EDC/HOBt mediated coupling of **4** with succinic anhydride according to Nguyen–Francis conditions [44] provided **5**, whose esterification with **1** under Steglich conditions [45,46,47] finally yielded **6**, albeit in a reduced yield of 42%.

**Scheme 2:**
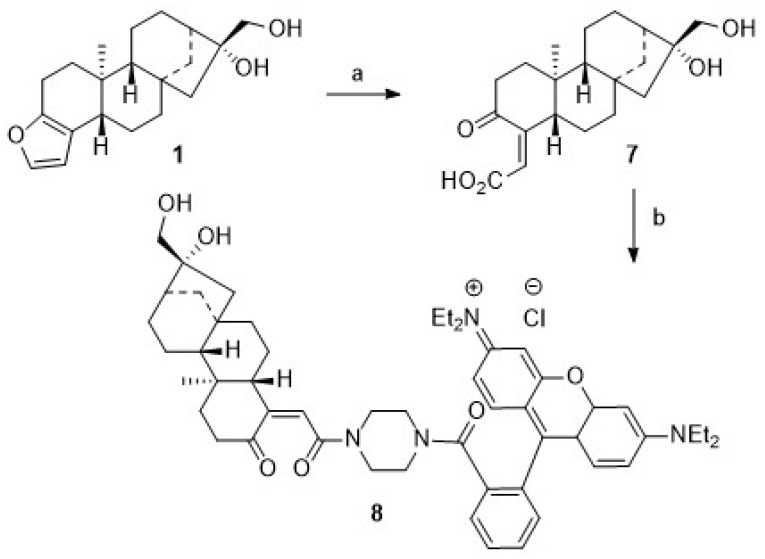
Reactions and conditions: (a) BAIB, H_2_O/CAN (1:0.5), 0 °C, TEMPO, 2 h, 81%; (b) DCM, EDC, HOBt, 20 °C, 15 min, then **4** (from Scheme 1) EA, microwaves (5 h, 55 °C, 1200 rpm), 59%.

We assumed that some more stable rhodamine B conjugates could be obtained from derivatives of **1**. Hence, attempts to oxidize the primary hydroxyl group of **1** were undertaken. Oxidation reactions of **1** using Jones [48] or Cornforth conditions [49] failed; the oxidation with Dess–Martin reagent [50,51,52,53,54] or TEMPO/NCS [55,56] or Pd/C/NaHCO_3_ and oxygen failed [57], too. In all of these reactions, vast deterioration of the starting material as well as many side reactions were observed. Reaction of **1** with TEMPO/BAIB according to Margarita and Piancatelli [55] in acetonitrile/water, however, led to the formation of product **7** (Scheme 2) in good yields. Close inspection of the analytical data of **7** revealed that the furan moiety was not intact any longer. Compound **7** was isolated as a colorless solid whose two carbonyl groups were detected in the ^13^C NMR spectrum at δ = 173.0 and 171.0 ppm, respectively. Carbons C-4 and C-18 were detected in the ^13^C NMR spectrum at δ = 104.9 and 112.3 ppm. The corresponding signals for the C = O moieties in the IR spectrum were located at ν = 1763 cm^−1^ and 1723 cm^−1^. Oxidative cleavage of furans, however, is not unprecedented. A similar ring opening reaction has been reported upon treatment of **2** at low pH [58].

**Scheme 3:**
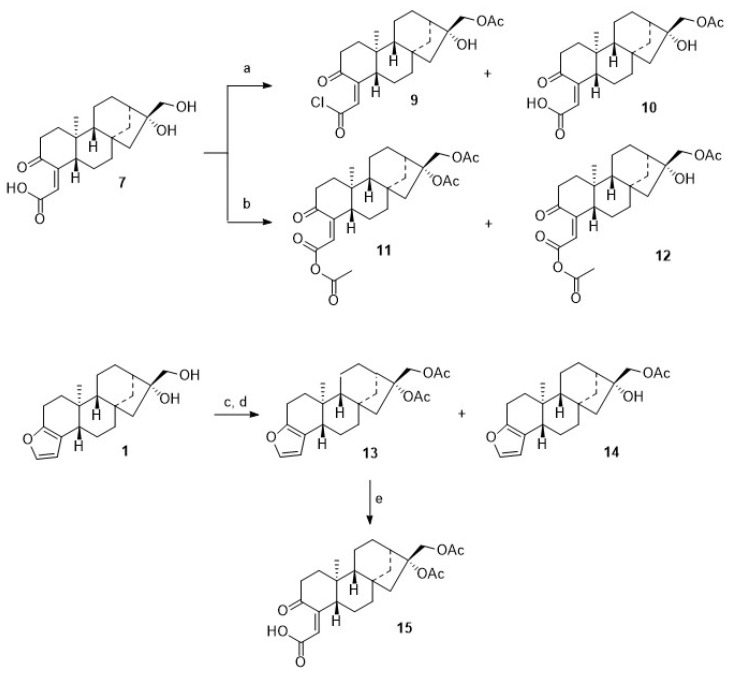
Reactions and conditions: (a) DCM, TEA, AcCl, 12 h, 20 °C, 37% of **9** and 50% of **10**; (b) pyridine, DMAP, Ac_2_O, 50 °C, 4.5 h, 64% of **11** and 6% of **12**; (c) DCM, AcCl, TEA, 12 h, 20 °C, 59% of **13** and 12% of **14**; (d) Ac_2_O, pyridine, 24 h, 20 °C, 94% of **13**; (e) BAIB, H_2_O, H_2_O/ACN (1:0.5), 0 °C, TEMPO, 12 h, 99%.

Microwave-assisted coupling of **7** with **4** in the presence of EDC/HOBt/TEA gave **8**. Interestingly enough, upon treatment of **7** with acetyl chloride in the presence of TEA, a mixture of **9** and **10** was obtained, while from the reaction of **7** with acetic anhydride/pyridine/DMAP (cat.), a mixture of **11** and **12** was isolated (Scheme 3). These mixtures were easily separated by chromatography, and pure products **9**–**12** were isolated, albeit in somewhat diminished yields. These reactions proved to be very sensitive to traces of moisture; this is most likely the reason why, even with an excess of acylating reagent, no complete conversion to the diacetylated product could be observed. On the other hand, it was possible to obtain monoacetylated products. However, acetylation of **1** with acetic anhydride (distilled over P_4_O_10_) and pyridine (freshly distilled over barium oxide) at 20 °C for 24 h with strict exclusion of moisture gave 94% of the diacetate **13**, thus paralleling previous results obtained by Hauptmann and Franca [59].

From the acetylation of **1** with acetyl chloride, however, diacetylated cafestol **13** and monoacetate **14** were isolated. Oxidation of **13** with TEMPO/BAIB afforded, again, cleavage of ring A, and **15** was obtained in an almost quantitative yield.

**Scheme 4:**
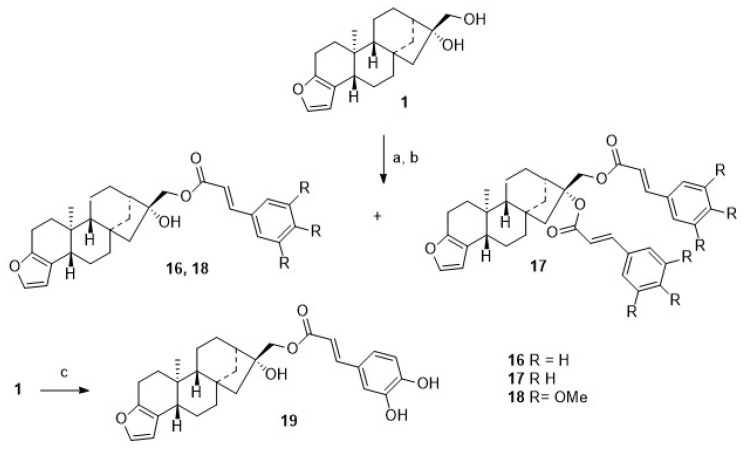
Reactions and conditions: (a) DCM, TEA, cinnamoyl chloride, 12 h, reflux, then 12 h, 20 °C, 34% of **16** and 16% of **17**; (b) DCM, TEA, 3,4,5-trimethoxy-cinnamoyl chloride, 12 h, reflux, then 12 h, 20 °C, 75%; (c) DCM, TEA, caffeoyl chloride, 12 h, reflux, then 12 h, 20 °C, 73%.

For comparison, **1** was acylated with cinnamic acid chloride (Scheme 4), and monoacylated **16** was obtained together with diacylated **17**. From the reaction of **1** with either 3,4,5-trimethoxy-cinnamic acid chloride, monoester **18** was obtained, as from caffeic acid chloride, monoester **19** was formed, too.

To assess their cytotoxicity, selected compounds were subjected to SRB assays employing some representative human tumor cell lines (MCF7, A2780) and non-malignant fibroblasts (NIH 3T3). The results from these assays are compiled in Table 2. MCF7 breast adenocarcinoma cells have been used since they are the most studied human breast cancer cell lines, with breast cancer being by far the most common cancer in women nowadays. A2780 ovarian cancer cells are commonly used as a model for ovarian cancer. This type of cancer is the 8^th^ most common cancer in women. SRB assays can safely be used for the cytotoxic assessment of these compounds, since there is a sufficient difference in their respective UV/fluorescence spectra (λ_SRB_ = 510 nm; for the rhodamine B conjugates, λ = 560–570 nm).

These results show that the parent compounds, such as compounds **7** and **16**–**19**, did not exhibit significant cytotoxicity in the SRB assay. Compounds **3**–**5** and **8** showed increased cytotoxicity in all the cell lines tested, but the selectivity (ratio of IC_50_ values between the respective malignant and non-malignant cell lines) was extremely poor. It is noteworthy that compound **6** has sufficient cytotoxicity against the malignant cell lines MCF7 and A2780 but is significantly less cytotoxic against the non-malignant fibroblast NIH 3T3. Prior to biological testing, the stability of compounds in the medium used for the SRB assay was investigated. Solutions of the compounds (pH 7.5, 37 °C, 72 h) showed no or minimal decomposition (<5%; by HPLC).

These results confirm once again that the cytotoxic effect of terpene–rhodamine B conjugates is highly dependent on the type of terpene used. It appears to be confirmed [23,24,25,26,27,28,29,30,31] that the cytotoxic effect, as well as the selectivity between malignant and non-malignant cells, is particularly pronounced with polyacetylated pentacyclic triterpene-conjugates, whereas significantly reduced cytotoxicity and reduced selectivity are observed for their diterpenoid analogs. This especially holds true for hybrids holding a rhodamine B unit attached to a triterpene scaffold (for example, betulinic acid, oleanolic acid, ursolic acid, asiatic acid, madecassic acid, corosolic acid, platanic or boswellic acid) as compared to several analogs holding, for example, a dehydroabietyl or (iso)-steviosyl moiety.

## 3. Discussion

Coffee, one of the world’s most consumed beverages, originates from the Kaffa region in Ethiopia, with its usage traced back to 900 AD. Recently, unroasted “green coffee” has gained attention for purported effects, including weight loss and reduced risks of cancer and metabolic diseases, possibly due to its rich content of diterpenes, such as cafestol and kahweol.

Our study focused on isolating and characterizing these diterpenes from green coffee beans, particularly cafestol (**1**) and kahweol (**2**). Extraction and saponification methods were optimized, and separation challenges were addressed through the use of AgNO_3_-impregnated silica gel. NMR spectroscopic techniques, including advanced 2D methods (e.g., HSQC, HMBC, (IN)ADEQUATE), enabled full structural assignment of both compounds in CDCl_3_ as solvent. Previously, a full assignment (resulting from a series of several 2D NMR experiments but no (IN)ADEQUATE experiments) in CD_3_OD was published by de Luca et al. [60] in 2009.

Due to the instability of kahweol in solution, the focus shifted to cafestol for the synthesis of rhodamine B conjugates. Initial attempts at direct ester coupling produced unstable products. Improved stability and yields were achieved using modified rhodamine B derivatives and optimized coupling strategies. Additional derivatives were synthesized via oxidation and acetylation reactions, although oxidative cleavage of the furan moiety presented challenges.

Biological evaluation using SRB assays on human cancer cell lines (MCF7, A2780) and non-malignant fibroblasts (NIH 3T3) revealed that parent diterpenes and many simple derivatives lacked cytotoxic activity. However, selected cafestol–rhodamine B conjugates exhibited significant cytotoxic effects, particularly compound **6**, which demonstrated a favorable selectivity profile. The findings support the hypothesis that the bioactivity of rhodamine B conjugates is contingent on the terpene scaffold, with diterpenoid conjugates showing limited efficacy compared to their triterpenoid counterparts.

This study underscores the potential of diterpene-based conjugates as chemotherapeutic agents, while highlighting the need for further structural optimization to enhance both potency and selectivity.

## 4. Materials and Methods

Reagents were bought from commercial suppliers and used without further purification. The solvents were dried according to usual procedures. TLC was performed on silica gel (Macherey-Nagel, detection with UV absorption; Macherey-Nagel, Düren, Germany). Melting points were measured with a Büchi M-565 instrument (Büchi Labortechnik, Flawill, Switzerland). NMR spectra were recorded using VARIAN spectrometers (Varian Germany, Darmstadt, Germany) at 27 °C (δ given in ppm; *J* in Hz; typical experiments for assignments: ^13^C APT, HMBC, HSQC). ASAP-MS spectra were taken on an Advion (Advion, Ithaca, NY, USA) expression CMS-L with an ASAP/APCI ion source (capillary voltage, 150 V; capillary temperature, 220 °C; voltage of the ion source, 15 V; APCI source temperature, 300 °C with 5 μA). IR spectra were recorded on a Perkin-Elmer Spectrum Two (UATR Two Unit, Perkin-Elmer GmbH, Rodgau, Germany). TLC plates (SiO_2_, F_254_ from Macherey-Nagel) were impregnated with AgNO_3_ (10% followed by drying at 110 °C). AgNO_3_-impregnated silica gel was freshly prepared from silica gel (180 g, 0.040–0.063 mm, Merck) and AgNO_3_ (20 g in 40 mL of water), followed by drying at 110 °C for 1 h. Green coffee beans (*Coffea arabica*, Lagona, Brazil) were obtained from a local supplier. Cytotoxic activities of the compounds were analyzed using the SRB cytotoxicity assay. Cells were seeded in 96-well plates and, after 24 h, treated with serial dilutions of compounds for 72 h. All subsequent steps were performed according to the previously described SRB assay protocol [24,25,27,29,61]. Dose–response curves and calculations of IC_50_ values, including standard deviations, were carried out using GraphPad Prism (version 8) (https://www.graphpad.com, accessed on 17 March 2025).

The purity, as well as the structural integrity of the compounds, was primarily confirmed through detailed NMR analysis. The location and intensity of the signals are perfectly in line with expectations for these structures. The ^1^H and ^13^C NMR spectra exhibited clean, well-resolved signals without any extra peaks, baseline noise, or signs of contamination, thus indicating a high level of purity. Additional ESI-MS was performed, and the observed quasi-molecular ions (as well as their isotopic pattern) were consistent with the proposed structure. Microanalysis was performed for all the compounds and provided satisfactory results. For the compounds to be subjected to biological screening, extra HPLC measurements were performed (column: Zorbax Eclipse XDB-C18 (from a local supplier, Agilent, Waldbronn, Germany), 150 × 4.6 mm, mobile phase MeOH/H_2_O 68:32 (*v/v*), flow rate 0.7 mL/min). Thereby, for cafestol, a retention time of 12.9 min was determined; for kahweol—11.7 min. The purity of all compounds was > 95% (by HPLC), except for cafestol and kahweol (purity > 99.5%, by HPLC).

### 4.1. Cafestol (***1***, CAS: 469-83-0) and Kahweol (***2***, CAS: 6894-43-5) by Extraction

Green coffee beans were frozen in liquid nitrogen and crushed. The coffee powder (175 g) was extracted in a Soxhlet apparatus (12 h, 95 °C) with MTBE (1 L); the solvent was removed under diminished pressure. The remaining oil (greenish, 16.89 g) was added to an ethanolic solution of KOH (10%, 500 mL), and the mixture was heated under reflux for 2 h. The solvents were removed under diminished pressure, the residue was dissolved in water (300 mL), and an aqueous solution of NaCl (10%, 85 mL) was added, followed by extraction with ether (3 × 150 mL). The organic phases were combined, and after removal of the solvent under diminished pressure, a residue was obtained, that was subjected to chromatography (silica gel, *n*-hexane/ethyl acetate, 3:7) to yield a mixture of **1** and **2** (1.56 g, 0.89% of dry weight) as a yellow solid. Re-chromatography of this mixture on impregnated silica gel (*n*-hexane/ethyl acetate, 3:7) gave **1** (315 mg) and **2** (343 mg), each as a white solid. Repetition of this experiment (thrice) always afforded yields (**1** + **2**) between 1.45 and 1.65 g, respectively.

As an alternative, spent coffee grounds were used. Thereby, from 200 g of this material (Tre Forze! beans (from roastmarket.de, Frankfurt, Germany), previously used to prepare coffee, dried at 70–80 °C to remove moisture, then stored in airtight containers at 5 °C for later use), a mixture of **1** and **2** (1.25 g, 0.63% of dry weight) was obtained and separated as described above.

Data for **1**: m.p. 157–161 °C (lit. [62], 158–159 °C); R_f_ = 0.15 (silica gel, hexanes/ethyl acetate, 2:1); R_f_ = 0.26 (impregnated silica gel, hexanes/ethyl acetate, 2:1); ${\left[\alpha \right]}_{D}^{20}$ = −117.5° (*c* = 0.21, CHCl_3_), lit. [63], ${\left[\alpha \right]}_{D}^{20}$ = −119° (*c* 0.06, MeOH); IR (KBr): ν = 3551 w, 3402 br, 2916 m, 2854 m, 1454 m, 1045 s cm^−1^; UV–vis (CHCl_3_): λ_max_ (log ε) = 240 nm (3.67); for ^1^H and ^13^C NMR: cf. Table 1; MS (ESI, MeOH): *m*/*z* (%) = 494.3 ([3M+Ca]^2+^, 100%), 339.2 ([M+Na]^+^, 48%), 317.2 ([M+H]^+^ 14%); analysis: calculated for C_20_H_28_O_3_ (316.44): C 75.91, H 8.92; found: C 75.77, H 9.09.

Data for **2**: m.p. 123–126 °C (lit. [18], 88–90 °C); R_f_ = 0.18 (impregnated silica gel, toluene/ethyl acetate/formic acid/*n*-heptane, 80:26:5:10); ${\left[\alpha \right]}_{D}^{20}$ = −269.3° (*c* = 0.20, MeOH), lit. [18], −270° (*c* 1.0, MeOH); IR (KBr): ν = 3382 m, 2926 s, 2864 m, 1670 w, 1468 m, 1448 m, 1173 m, 1132 m, 1042 s, 1015 s cm^−1^; UV–vis (CHCl_3_): λ_max_ (log ε) = 310 nm (3.80); ^1^H and ^13^C NMR: cf. Table 1; MS (ESI, MeOH): *m*/*z* (%) = 297.1 ([M+H_2_O–H]^+^, 29%), 315.1 ([M+H]^+^, 7%), 385.2 ([M+K+MeOH]^+^, 100%); analysis: calculated for C_20_H_26_O_3_ (314.42): C 76.40, H 8.33; found: C 76.19, H 8.52.

### 4.2. Cafestol (***1***) by Hydrogenation from a Mixture of ***1*** and ***2***

To a solution of **1** and **2** (793 mg; ratio 1:2 = 1:1) in dry methanol (25 mL), Pd/CaCO_3_ (5% Pb; catalytic amount) was added, and the mixture was hydrogenated at 20 °C (4 h, 3.5 bar; TLC showed completion of the reaction). The mixture was filtered through Celite, the solvent was removed, and **1** (778 mg, 98%) was obtained as a white solid; analytical data as above.

### 4.3. [9-[2-(Cafest-17-yl)carboxyphenyl]-3,6-bis(diethylamino)]-xanthylium Chloride (***3***)

To an ice-cold solution of **1** (100 mg, 0.32 mmol) in dry DCM (10 mL), DMAP (10 mg, 0.08 mmol) and rhodamine B (169 mg, 0.35 mmol) were added, followed by the addition of DCC (79 mg, 0.38 mmol). The mixture was stirred at 20 °C for 1 d. Usual aqueous work-up, followed by chromatography (silica gel, CHCl_3_/MeOH, 9:1.2), gave **3** (136 mg, 57.3%) as a dark violet solid; m.p. 183–185 °C; R_f_ = 0.38 (silica gel, CHCl_3_/MeOH, 9:1.2); IR (ATR): ν = 1716 m, 1647 m, 1586 s, 1465 m, 1410 s, 1333 s, 1271 s, 1245 s, 1178 s, 1129 s, 1072 s cm^−1^; ^1^H NMR (500 MHz, CD_3_OD): δ = 8.43–8.37 (*m*, 1H, 6′-H), 7.96–8.82 (*m*, 3H, 11′-H + 3′-H), 7.47 (*d*, *J* = 7.4 Hz, 1H, 5′H), 7.29 (*d*, *J* = 1.7 Hz, 1H, 19-H), 7.25–7.13 (*m*, 1H, 4-H), 7.13–7.04 (*m*, 2H, 10‘-H), 7.03 (*d*, *J* = 1.8 Hz, 2H, 13‘-H), 6.24 (*d*, *J* = 1.8 Hz, 1H, 18-H), 4.23 (*d*, *J* = 2.3 Hz, 1H, 17-H_a_), 4.20 (*m*, 1H, OH), 4.19 (*d*, *J* = 4.0 Hz, 1H, 17-H_b_), 3.77–3.68 (*m*, 8H, 15‘-H_a_ + 15‘-H_b_), 2.60 (*d*, *J* = 8.2 Hz, 2H, 2-H_a_ + 2-H_b_), 2.30–2.21 (*m*, 1H, 5-H), 2.10–2.02 (*m*, 1H, 1-H_a_), 1.93 (*d*, *J* = 11.6 Hz, 1H, 14-H_a_), 1.87–1.77 (*m*, 1H, 12-H_a_), 1.77–1.67 (*m*, 1H, 13-H), 1.70–1.59 (*m*, 3H, 7-H_a_ + 6-H_a_ + 11-H_a_), 1.60–1.40 (*m*, 4H, 14-H_b_ + 7-H_b_ + 12-H_b_ + 11-H_b_), 1.41–1.30 (*m*, 1H, 6-H_b_), 1.35 (*s*, 12H, 16′-H), 1.29–1.15 (*m*, 1H, 1-H_b_), 1.14–1.02 (*m*, 3H, 15-H_a_ + 15-H_b_ + 9-H), 0.81 (*s*, 3H, 20-H) ppm; ^13^C NMR (100 MHz, CD_3_OD): δ = 165.9 (C-1′), 157.9 (C-14′), 155.8 (C-8′), 155.7 (C-12′), 148.3 (C-3), 140.5 (C-19), 133.2 (C-2′), 132.7 (C-11′), 132.5 (C-7′), 130.8 (C-6′), 130.8 (C-4′), 130.2 (C-10′), 130.0 (C-3′), 119.8 (C-4), 114.0 (C-10′), 113.6 (C-9′), 107.6 (C-18), 95.9 (C-13′), 79.0 (C-16), 69.4 (C-17), 52.6 (C-15), 52.1 (C-9), 45.4 (C-15‘), 45.4 (C-13), 44.3 (C-8), 44.2 (C-5), 40.6 (C-7), 38.3 (C-10), 37.5 (C-14), 35.5 (C-1), 25.5 (C-6), 22.7 (C-12), 20.0 (C-2), 18.3 (C-11), 12.2 (C-16′), 11.4 (C-20) ppm; MS (ESI, MeOH): *m*/*z* (%) = 741.4 ([M-Cl]^+^, 100%); analysis: calculated for C_48_H_59_N_2_O_5_Cl (779.46): C 73.97, H 7.63, N 3.59; found: C 73.65, H 7.91, N 3.37.

### 4.4. 3,6-Bis(diethylamino)-9-[2-(1-piperazinylcarbonyl)phenyl]xanthylium Chloride (***4***, CAS: 608136-11-4)

This compound was prepared as previously reported [44]; m.p. > 250 °C (lit. [44], m.p. > 250 °C).

### 4.5. 9-[2[[4-(3-Carboxy-1-oxopropyl)-1-piperazinyl]carbonyl]phenyl]-3,6-bis(diethylamino)-xanthylium Chloride (***5***, CAS: 608136-12-5)

To a solution of **4** (100 mg, 0.20 mmol) in dry DCM (10 mL), TEA (0.04 mL, 0.30 mmol), DMAP (24 mg, 0.20 mmol), and succinic anhydride (20 mg, 0.20 mmol) were added, and the mixture was stirred at 20 °C for 1 d. Usual aqueous work-up followed by chromatography (silica gel, CHCl_3_/MeOH, 9:1) afforded **5** (97 mg, 75%) as a violet solid; m.p. > 350 °C (lit. [44], 166–168 °C); R_f_ = 0.25 (silica gel, CHCl_3_/MeOH, 9:1.7); IR (ATR): ν = 3326 s, 1954 s, 1417 s, 1347 s, 1279 m, 1182 m cm^−1^; ^1^H NMR (500 MHz, CD_3_OD): δ = 7.81–7.77 (*m*, 2H, 5-H + 6-H), 7.73–7.71 (*m*, 1H, 3-H), 7.57–7.51 (*m*, 1H, 4-H), 7.30 (*dd*, *J* = 9.5, 4.2 Hz, 2H, 11-H), 7.09 (*d*, *J* = 9.3 Hz, 2H, 10-H), 6.98 (*d*, *J* = 2.5 Hz, 2H, 13-H), 3.71 (*q*, *J* = 7.0 Hz, 8H, 15-H_a_ + 15-H_b_), 3.48–3.40 (*m*, 8H, 19-H_a_ + 19-H_b_ + 17-H_a_ + 17-H_b_ + 20-H_a_ + 20-H_b_ + 18-H_a_ + 18-H_b_), 2.59 (*dd*, *J* = 21.2, 6.1 Hz, 4H, 22-H_a_ + 22-H_b_ + 23-H_a_ + 23-H_b_), 1.33 (*d*, *J* = 6.7 Hz, 12H, 16-H) ppm; ^13^C NMR (100 MHz, CD_3_OD): δ = 174.7 (C-24), 171.2 (C-21), 168.1 (C-1), 157.8 (C-14), 155.8 (C-8), 155.8 (C-12), 135.1 (C-2), 131.8 (C-11), 130.3 (C-4), 129.9 (C-5), 129.9 (C-6), 127.9 (C-7), 127.5 (C-3), 114.0 (C-10), 113.4 (C-9), 95.9 (C-13), 46.9 (C-18), 44.7 (C-20), 41.1 (C-17), 37.9 (C-19), 28.9 (C-23), 27.5 (C-22), 11.4 (C-16) ppm; MS (ESI, MeOH): *m*/*z* (%) = 611.5 ([M-Cl]^+^, 100%); analysis: calculated for C_36_H_43_N_4_O_5_Cl (647.20): C 66.81, H 6.70, N 8.66; found: C 66.51, H 6.93, N 8.29.

### 4.6. N-[6-(Diethylamino)-9-[2-[[4-[4-(cafestyl)oxy]-4-oxobutanoyl]piperazin-1-yl]carbonyl)phenyl-3H-xanthen-3-ylidene]-N-ethyl-ethanaminium Chloride (***6***)

To an ice-cold solution of **5** (530 mg, 0.82 mmol) in dry DCM (25 mL), EDC (0.242 g, 1.54 mmol), DIPEA (0.27 mL, 1.59 mmol), and **1** (0.26 g, 0.82 mmol), as well as DMAP (catalytic amounts) were added, and the mixture was stirred at 20 °C for 3 h. Usual aqueous work-up, followed by chromatography (silica gel, CHCl_3_/MeOH, 9:1), gave **6** (0.33 g, 42%) as a violet solid; m.p. 218–221 °C; R_f_ = 0.50 (CHCl_3_/MeOH, 4:1); IR (ATR): ν = 2928 br, 1731 w, 1568 s, 1411 m, 1333 s, 1272 m, 1245 m, 1178 s, 1131 m, 1072 m, 1006 m cm^−1^; ^1^H NMR (500 MHz, CD_3_OD): δ = 7.78–7.69 (*m*, 3H, 6′-H + 7′-H + 15-H), 7.52–7.50 (*m*, 2H, 9′-H, 14-H), 7.31–7.19 (*m*, 2H, 10′-H, 19-H), 7.10–7.06 (*m*, 1H, 13′-H), 6.97–6.96 (s, 1H, 17′-H), 6.20–6.19 (d, 1H, 18-H), 4.31–4.14 (*m*, 1H, 17-H), 3.73–3.31 (*m*, 10H, 14-H + 22-H_a_ + 22-H_b_ + 24-H_a_ +21-H_a_ + 21-H_b_ + 23-H_a_ +23-H_b_ + 19′-H), 2.69–2.54 (t, 6H, 2′-H_a_ +2′-H_b_ + 3′-H_a_ + 3′-H + 2-H_a_ + 2-H), 2.24–1.90 (*m*, 4H, 5-H + 13-H +1-H_a_ + 1-H_b_), 1.82–1.42 (*m*, 8H, 6-H_a_ + 6-H_b_ + 11-H_a_ + 11-H_b_ + 7-H_a_ + 7-H_b_ + 12-H_a_ + 12H), 1.37–1.25 (*m*, 3H, 20′-H_a_ + 20′-H_c_), 0.97–0.80 (*m*, 3H, 20-H_a_ + 20-H_b_ + 20-H_c_) ppm; ^13^C NMR (126 MHz, CD_3_OD): δ = 174.7 (C-1′), 172.6 (C-4′), 169.6 (C-5′), 159.2 (C-12′), 157.2 (C-16′), 149.7 (C-3), 141.8 (C-19), 136.5 (C-11′), 133.2 (C-10′), 132.3 (C-6′), 131.8 (C-9′ + C-14′), 131.3 (C-8′), 128.9 (C-7′ + C-15′), 121.3 (C-4), 115.4 (C-13′), 109.1 (C-18), 97.4 (C-17′), 80.9 (C-16), 69.5 (C-17), 54.1 (C-15), 53.5 (C-9), 46.9 (C-22′ + C-24′), 45.8 (C-19′), 45.5 (C-5 + C-13), 44.3 (C-8), 42.9 (C-21′ + C-23), 42.0 (C-7), 39.8 (C-10), 39.1 (C-14), 36.9 (C-1), 30.11 (C-3′), 28.8 (C-2′), 27.1 (C-12), 24.1 (C-6), 21.4 (C-2), 19.9 (C-11), 13.7 (C-20), 12.9 (C-20′) ppm; MS (ESI, MeOH): *m*/*z* (%) = 927.5 ([M-Cl]^+^, 46%); analysis: calculated for C_57_H_75_N_4_O_7_Cl (963.68): C 71.04, H 7.84, N 5.81; found: C 70.63, H 8.14, N 5.55.

### 4.7. (16,17-Dihydroxy-3-oxo-4,4-dinorkauran-4-ylidene)-acetic Acid (***7***)

To an ice-cold solution of BAIB (384 mg, 1.19 mmol) in acetonitrile/water (1:0.5, 20 mL), TEMPO (56 mg, 0.48 mmol) and **1** (150 mg, 0.47 mmol) were added, and the mixture was stirred for another 2 h. Usual aqueous work-up, followed by chromatography (silica gel, ethyl acetate/chloroform, 4:1), gave **7** (130 mg, 81%) as a white solid; m.p. 227–229 °C; R_f_ = 0.24 (silica gel, ethyl acetate/chloroform, 4:1); ${\left[\alpha \right]}_{D}^{20}$ = −178.9° (*c* = 0.28, DMSO); UV–vis (CHCl_3_): λ_max_ (log ε) = 262 nm (2.5); IR (KBr): ν = 3424 s, 2924 m, 2866 w, 1764 s, 1724 m, 1656 m, 1452 w, 1196 w, 1040 m cm^−1^; ^1^H NMR (400 MHz, DMSO-d_6_): δ = 7.22 (s, 1H, 19-OH), 5.72 (*d*, *J* = 1.5 Hz, 1H, 18-H), 4.34 (*t*, *J* = 5.6 Hz, 1H, 17-OH), 3.90 (s, 1H, 16-OH), 3.53 (*dd*, *J* = 10.9 Hz, 1H, 17-H_a_), 3.42 (*dd*, *J* = 11.1, 5.2 Hz, 1H, 17-H_b_), 2.20 (*d*, *J* = 10.9 Hz, 1H, 5-H), 2.16–2.07 (*m*, 1H, 2-H_a_), 1.91 (s, 1H, 13-H), 1.84–1.64 (*m*, 3H, 14-H_a_ + 1-H_a_ + 2-H_b_), 1.62–1.45 (*m*, 9H, 11-H_a_ + 11-H_b_ + 6-H_a_ + 6-H_b_ + 12-H_a_ + 7-H_a_ + 7-H_b_ + 14-H_b_+ 15-H_a_), 1.41–1.27 (*m*, 2H, 12-H_b_ + 15-H_b_), 1.26–1.12 (*m*, 2H, 9-H + 1-H_b_), 0.77 (s, 3H, 20-H) ppm; ^13^C NMR (100 MHz, DMSO-d_6_): δ = 173.0 (C-3), 171.0 (C-19), 112.3 (C-18), 104.9 (C-4), 80.9 (C-16), 65.7 (C-17), 53.4 (C-9), 53.2 (C-15), 46.9 (C-5), 45.0 (C-13), 44.2 (C-8), 43.4 (C-10), 39.4 (C-7), 37.7 (C-14), 35.6 (C-1), 34.3 (C-2), 27.3 (C-12), 21.9 (C-6), 19.2 (C-11), 14.6 (C-20) ppm; MS (ESI, MeOH): *m*/*z* (%) = 349.1 ([M+H]^+^, 100%); analysis: calculated for C_20_H_28_O_5_ (348.43): C 68.94, H 8.10; found: C 68.65, H 8.31.

### 4.8. 9-[2-[[4-(16,17-Dihydroxy-3-oxo-4,4-dinorkauran-4-ylidene-acetoyl)-1-piperazinyl]carbonylphenyl]-3,6-bis(diethylamino)-xanthylium Chloride (***8***)

To a solution of **7** (120 mg, 0.34 mmol) in dry DCM (10 mL), EDC (132 mg, 0.69 mmol) and HOBt (93 mg, 0.96 mmol) were added, and the mixture was stirred at 20 °C for 15 min, followed by adding **4** (352 mg, 0.69 mmol) and TEA (0.19 mL, 1.38 mmol). Microwave-assisted stirring (5 h, 55 °C, 1200 rpm), followed by usual aqueous work-up and chromatography (silica gel, CHCl_3_/MeOH, 9:2 → 9:1.6), gave **8** (180 mg, 59%) as a violet solid; m.p. 212–214 °C, R_f_ = 0.46 (silica gel, CHCl_3_/MeOH, 9:1.7), IR (ATR): ν = 2928 w, 2630 m, 1585 vs, 1410 s, 1332 s, 1272 s, 1244 s, 1177 vs, 1129 s, 1071 s, 1007 m cm^−1^; ^1^H NMR (500 MHz, CD_3_OD): δ = 7.85–7.79 (*m*, 2H, 4′-H + 5′-H), 7.77–7.72 (*m*, 1H, 3′-H), 7.60–7.54 (*m*, 1H, 6′-H), 7.33 (*d*, *J* = 9.5 Hz, 2, 11-H), 7.12 (*dd*, *J* = 9.5, 2.3 Hz, 2H, 10′-H), 7.01 (*d*, *J* = 2.5 Hz, 2H, 13′-H), 5.96 (*d*, *J* = 2.3 Hz, 1H, 18-H), 3.79–3.70 (*m*, 9H, 15′-H_a_ + 15′-H_b_ + 17-H_a_), 3.68–3.62 (*m*, 1H, 17-H_b_), 3.50–3.38 (*m*, 9H, 18′-H_a_ + 18′-H_b_ + 17′-H_a_ + 17′-H_b_ + 19′-H_a_ + 19′-H_b_ + 20′-H_a_ + 20′-H_b_ + 10-H), 2.68–2.53 (*m*, 1H, 1-H_a_), 2.46 (*dd*, *J* = 17.3, 8.0 Hz, 1H, 1-H_b_), 2.26–2.14 (*m*, 1H, 14-H_a_), 2.10 (s, 1H, 13-H), 2.06–1.90 (*m*, 1H, 2-H_a_), 1.82–1.51 (*m*, 10H, 11-H_a_ + 11-H_b_ + 12-H_a_ + 12-H_b_ + 2-H_b_ + 6-H_a_ + 6-H_b_ + 7-H_a_ + 7-H_b_ + 15-H_a_), 1.52–1.40 (*m*, 2H, 15-H_b_ + 14-H_b_), 1.35 (*t*, *J* = 7.1 Hz, 12H, 16-H), 1.29 (*d*, *J* = 7.4 Hz, 1H, 9-H), 1.09 (s, 3H, 20-H) ppm; ^13^C NMR (100 MHz, CD_3_OD): δ = 172.9 (C-3), 169.7 (C-19), 169.6 (C-1), 159.3 (C-14′), 157.2 (C-8′), 157.0 (C-12′), 136.5 (C-2′), 133.2 (C-11′), 132.2 (C-7′), 131.8 (C-6′), 131.3 (C-4′), 131.3 (C-5′), 128.9 (C-3′), 124.8 (C-18), 115.4 (C-10), 114.9 (C-9′), 111.4 (C-4), 97.4 (C-13′), 82.8 (C-16), 66.8 (C-17), 54.7 (C-9), 53.4 (C-15), 50.6 (C-5), 48.5 (C-17′), 46.9 (C-15′), 46.9 (C-18′), 46.2 (C-13), 45.3 (C-8), 44.5 (C-20′), 43.0 (C-19′), 42.6 (C-10), 40.6 (C-7), 39.3 (C-14), 38.6 (C-1), 37.9 (C-2), 26.9 (C-12), 23.6 (C-6), 20.2 (C-11), 16.2 (C-20), 12.8 (C-16′) ppm; MS (ESI, MeOH): *m*/*z* (%) = 843.6 ([M-Cl]^+^, 100%); analysis: calculated for C_52_H_67_N_4_O_6_Cl (879.56): C 71.03, H 7.69, N 6.38; found: C 70.75, H 7.97, N 5.97.

### 4.9. (17-Acetyloxy-16-hydroxy-3-oxo-4,4-dinorkauran-4-ylidene)-acetyl Chloride (***9***) and (17-acetyloxy-16-hydroxy-3-oxo-4,4-dinorkauran-4-ylidene)-acetic Acid (***10***)

To an ice-cold solution of **7** (100 mg, 0.29 mmol) in dry DCM (15 mL), TEA (0.04 mL, 0.29 mmol) and acetyl chloride (0.075 mL, 1.06 mmol) were added, and the mixture was stirred overnight at 20 °C. Usual aqueous work-up, followed by chromatography, gave **9** (44 mg, 37%) and **10** (56 mg, 50%) each as a white solid.

Data for **9**: m.p. 165–166 °C; R_f_ = 0.58 (silica gel, hexanes/ethyl acetate, 3:7); ${\left[\alpha \right]}_{D}^{20}$ = −96.4° (0.32, CHCl_3_); UV–vis (CHCl_3_): λ_max_ (log ε) = 265 nm (2.6); IR (KBr): ν = 3512 w, 1798 s, 1765 s, 1731 vs, 1390 m, 1366 m, 1246 vs, 1230 s, 1183 s, 1087 m cm^−1^; ^1^H NMR (400 MHz, DMSO-d_6_): δ = 6.13 (*s*, 1H, 18-H), 4.39 (*s*, 1H, 16-OH), 4.18 (*d*, *J*= 11.0 Hz, 1H, 17-Ha), 4.05 (*d*, *J* = 11.4 Hz, 1H, 17-H_b_), 2.46 (*s*, 1H, 1-H_a_), 2.37 (*d*, *J* = 6.4 Hz, 1H, 5-H), 2.19 (*t*, *J* = 14.0 Hz, 1H, 1-H_b_), 2.02 (*s*, 3H, 22-H), 1.98 (*s*, 1H, 13-H), 1.87 (*m*, 2H, 2-H_a_, 14-H_a_), 1.59 (*m*, 8H, 11-H_a_ + 11-H_b_ + 6-H_a_ + 6-H_b_ + 14-H_b_ + 7-H_a_ + 7-H_b_ + 15-H_a_), 1.51–1.08 (*m*, 5H, 12-H_a_+ 12-H_b_ + 2-H_b_ + 15-H_b_ + 9-H), 0.78 (*s*, 3H, 20-H) ppm; ^13^C NMR (100 MHz, DMSO-d_6_): δ = 173.5 (C-3), 171.1 (C-21), 169.3 (C-19), 121.7 C-18), 101.4 (C-4), 78.6 (C-16), 68.3 (C-17), 53.2 (C-15), 52.8 (C-9), 46.6 (C-5), 45.3 (C-13), 44.2 (C-8), 43.4 (C-10), 39,4 (C-7), 37.6 (C-14), 37.3 (C-1), 34.9 (C-2), 25.9 (C-12), 21.7 (C-6), 21.3 (C-22), 18.9 (C-11), 14.6 (C-20) ppm; MS (ESI, MeOH): *m*/*z* (%) = 409.1 ([M+H]^+^, 100%); analysis: calculated for C_22_H_29_O_5_Cl (408.92): C 64.62, H 7.15; found: C 64.36, H 7.41.

Data for **10**: m.p. 96–97 °C; R_f_ = 0.35 (silica gel, hexanes/ethyl acetate, 3:7): ${\left[\alpha \right]}_{D}^{20}$ = −167.1° (*c* = 0.31, CHCl_3_); UV–vis (CHCl_3_): λ_max_ (log ε) = 265 nm (2.5); IR (KBr): ν = 2931 m, 2866 w, 1733 i/s, 1656 w, 1238 s, 1222 s, 1039 s cm^−1^; ^1^H NMR (400 MHz, DMSO-d_6_): δ = 7.23 (*s*, 1H, 19-OH), 5.72 (*d*, *J* = 1.5 Hz, 1H, 18-H), 4.37 (s, 1H, 16-OH), 4.18 (*d*, *J* = 11.3 Hz, 1H, 17-H_a_), 4.05 (*d*, *J* = 10.0 Hz, 1H, 17-H_b_), 2.21 (*d*, *J* = 11.1 Hz, 2H, 13-H_a_ + 13-H_b_), 2.16–2.03 (*m*, 1H, 2-H_a_), 2.02 (*s*, 3H, 22-H), 1.99 (s, 1H, 5-H), 1.83 (*d*, *J* = 11.3 Hz, 1H, 14-H_a_), 1.79–1.57 (*m*, 4H, 1-H_a_ + 2-H_b_ + 14-H_b_ + 15-H_a_), 1.59–1.35 (*m*, 9H, 11-H_a_+ 11-H_b_ + 6-H_a_ + 6-H_b_ + 7-H_a_ + 7-H_b_ + 12-H_a_ + 12-H_b_ + 15-H_b_), 1.25 (*d*, *J* = 8.1 Hz, 1H, 9-H), 1.22–1.12 (*m*, 1H, 1-H_b_), 0.78 (*s*, 3H, 20-H) ppm; ^13^C NMR (100 MHz, DMSO-d_6_): δ = 173.0 (C-3), 171.1 (C-21), 171.0 (C-19), 112.3 (C-18), 104.9 (C-4), 78.6 (C-16), 68.3 (C-17), 53.2 (C-15), 53.2 (C-9), 46.9 (C-13), 45.4 (C-5), 44.4 (C-8), 43.4 (C-10), 39.4 (C-7), 37.6 (C-14), 35.6 (C-1), 34.2 (C-2), 26.0 (C-12), 21.8 (C-6), 21.3 (C-22), 19.0 (C-11), 14.5 (C-20) ppm; MS (ESI, MeOH): *m*/*z* (%) = 391.1 ([M+H]^+^, 100%); analysis: calculated for C_22_H_30_O_6_ (390.48): C 67.67, H 7.74; found: C 67.40, H 7.98.

### 4.10. 16,17-Diacetoxy-3-oxo-4,4-dinorkauran-4-ylidene Acetic Acid Anhydride (***11***) and 17-Acetoxy-16-hydroxy-3-oxo-3,4-dinorkauran-4-ylidene Acetic Anhydride (***12***)

To a solution of **7** (200 mg, 0.57 mmol) in dry pyridine (5 mL), a catalytic amount of DMAP and acetic anhydride (0.27 mL, 2.87 mmol) were added, and stirring at 50 °C was continued for 4.5 h. The solvents were distilled off, and the residue was subjected to chromatography (silica gel, hexanes/ethyl acetate, 6:4) to afford **11** (174 mg, 64%) and **12** (15 mg, 6%), each as a white solid.

Data for **11**: m.p. 224–225 °C; R_f_ = 0.44 (silica gel, hexanes/ethyl acetate, 6:4); ${\left[\alpha \right]}_{D}^{20}$ = −147.8° (*c* = 0.30, CHCl_3_); UV–vis (CHCl_3_): λ_max_ (log ε) = 263 nm (2.6); IR (ATR): ν = 1756 vs, 1743 s, 1726 s, 1371 s, 1256 vs, 1208 vs, 1170 s, 1145 m, 1042 s cm^−1^; ^1^H NMR (400 MHz, CDCl_3_): δ = 5.74 (*d*, *J* = 1.7 Hz, 1H, 18-H), 4.96 (*d*, *J* = 12.3 Hz, 1H, 17-Ha), 4.44 (*d*, *J* = 12.3 Hz, 1H, 17-H_b_), 2.61 (*ddd*, *J* = 14.5, 3.7, 2.4 Hz, 1H, 2-H_a_), 2.56–2.51 (*m*, 1H, 13-H), 2.06 (*s*, 3H, 26-H), 2.07 (*s*, 3H, 22-H), 2.06–2.03 (*m*, 2H, 5-H + 15-H_a_), 2.00 (*s*, 3H, 24-H), 2.01–1.96 (*m*, 1H, 14-H_a_), 1.89–1.68 (*m*, 3H, 1-H_a_ + 15-H_b_ + 2-H_b_), 1.67–1.60 (*m*, 7H, 11-H_a_+ 11-H_b_+ 12-H_a_ + 12-H_b_ + 6-H_a_ + 7-H_a_ + 7-H_b_), 1.59–1.48 (*m*, 2H, 6-H_b_ + 14-H_b_), 1.32 (*d*, *J* = 4.6 Hz, 1H, 9-H), 1.30–1.15 (*m*, 1H, 1-H_b_), 0.87 (s, 3H, 20-H) ppm; ^13^C NMR (100 MHz, CDCl_3_): δ = 170.8 (C-21), 170.7 (C-23), 169.8 (C-3), 196.6 (C-19), 168.3 (C-25), 114.0 (C-18), 104.4 (C-4), 90.0 (C-16), 63.2 (C-17), 53.0 (C-9), 51.1 (C-15), 47.2 (C-5), 44.1 (C-8), 43.1 (C-13), 43.1 (C-10), 39.5 (C-7), 37.5 (C-14), 35.2 (C-1), 33.0 (C-2), 25.4 (C-6), 22.4 (C-24), 21.7 (C-22), 21.5 (C-12), 20.8 (C-26), 19.1 (C-11), 14.5 (C-20) ppm; MS (ESI, MeOH): *m*/*z* (%) = 497.1 ([M+Na]^+^, 100%); analysis: calculated for C_26_H_34_O_8_ (474.55): C 65.81, H 7.22; found: C 65.43, H 6.87.

Data for **12**: m.p. 81–83 °C; R_f_ = 0.13 (silica gel, hexanes/ethyl acetate, 6:4); ${\left[\alpha \right]}_{D}^{20}$ = −120.9° (*c* = 0.10, CHCl_3_); ^1^H NMR (400 MHz, CDCl_3_): δ = 5.98 (*d*, *J* = 1.7 Hz, 1H, 18-H), 4.38 (*s*, 1H, 16-OH), 4.20 (*d*, *J* = 11.3 Hz, 1H, 17-H_a_), 4.05 (*d*, *J* = 7.0 Hz, 1H, 17-H_b_), 2.48–2.38 (*m*, 1H, 2-H_a_), 2.18–2.09 (*m*, 1H, 5-H), 2.06 (s, 3H, 24-H), 2.02 (s, 3H, 22-H), 2.03–1.94 (*m*, 1H, 13-H), 1.88–1.66 (*m*, 3H, 14-H_a_ + 1-H_a_ + 2-H_a_), 1.66–1.57 (*m*, 4H, 12-H_a_ + 14-H_b_ + 7-H_a_ + 15-H_a_), 1.59–1.35 (*m*, 8H, 11-H_a_ + 11-H_b_ + 12-H_b_ + 6-H_a_ + 6-H_b_ + 7-H_b_ + 14-H_b_ + 15-H_b_), 1.30 (*d*, *J* = 8.1 Hz, 1H, 9-H), 1.17 (*t*, *J* = 7.1 Hz, 1H, 1-H_b_), 0.81 (*s*, 3H, 20-H) ppm; ^13^C NMR (100 MHz, CDCl_3_): δ = 171.1 (C-21), 170.5 (C-3), 170.1 (C-19), 169.0 (C-23), 114.1 (C-18), 104.6 (C-4), 78.6 (C-16), 68.3 (C-17), 53.2 (C-15), 52.8 (C-9), 46.8 (C-5), 45.4 (C-13), 44.3 (C-8), 43.3 (C-10), 39.4 (C-7), 37.6 (C-14), 34.8 (C-1), 33.3 (C-2), 26.0 (C-6), 21.9 (C-24), 21.8 (C-12), 21.3 (C-22), 19.0 (C-11), 14.6 (C-20) ppm; MS (ESI, MeOH): *m*/*z* (%) = 455.1 ([M+Na]^+^, 100%); analysis: calculated for C_24_H_32_O_7_ (432.51): C 66.65, H 7.46; found: C 66.59, H 7.67.

### 4.11. 16,17-Di-O-acetyl-cafestol (***13***), 17-O-acetyl-cafestol (***14***)

Acetylation of **1** (500 mg, 1.58) in dry DCM (10 mL) with acetyl chloride (0.42 mL, 5.85) as described above, followed by usual aqueous work-up and chromatography (silica gel, hexanes/ethyl acetate, 4:1), furnished **13** (372 mg, 59%) and **14** (66 mg, 12%) each as a white solid.

Alternatively, **13** was prepared from **1** [64] in dry pyridine with Ac_2_O following Wettstein’s procedure in 94% yield [63].

Data for **13**: m.p. 144–145 °C; R_f_ = 0.65 (silica gel, hexanes/ethyl acetate, 4:1); ${\left[\alpha \right]}_{D}^{20}$ = −183.5° (*c* = 0.33, CHCl_3_), lit. [64], ${\left[\alpha \right]}_{D}^{20}$ = −185° (*c* = 0.5, CHCl_3_); UV–vis (CHCl_3_): λ_max_ (log ε) = 266 nm (2.9); IR (KBr): ν = 3424 m, 2938 s, 2856 m, 1742 vs, 1720 vs, 1500 w, 1456 m, 1374 s, 1268 vs, 1254 vs, 1228 s, 1126 w, 1038 s, 1012 m cm^−1^; ^1^H NMR (400 MHz, DMSO-d_6_): δ = 7.39 (*d*, *J* = 1.6 Hz, 1H, 19-H), 6.29 (*d*, *J* = 1.8 Hz, 1H, 18-H), 4.92 (*d*, *J* = 12.4 Hz, 1H, 17-H_a_), 4.33 (*d*, *J* = 12.4 Hz, 1H, 17-H_b_), 2.60–2.53 (*m*, 2H, 2-H_a_ + 2-H_b_), 2.42–2.36 (*m*, 1H, 5-H), 2.26–2.17 (*m*, 1H, 13-H), 2.02 (s, 3H, 22-H), 2.05–1.97 (*m*, 2H, 1-H_a_ + 14-H_b_) 1.92 (s, 3H, 24-H), 1.85 (*d*, *J* = 12.5 Hz, 2H, 15-H_a_ + 15-H_b_), 1.83–1.73 (*m*, 1H, 6-H_a_), 1.72–1.48 (*m*, 6H, 11-H_a_ +11-H_b_ + 12-H_a_+ 12-H_b_ + 7-H_a_ + 7-H_b_), 1.47–1.34 (*m*, 2H, 6-H_b_ + 14-H_b_), 1.26–1.14 (*m*, 2H, 1-H_b_ + 9-H), 0.76 (s, 3H, 20-H) ppm; ^13^C NMR (100 MHz, DMSO-d_6_): δ = 170.7 (C-23), 170.5 (C-21), 148.5 (C-3), 141.4 (C-19), 120.2 (C-4), 108.9 (C-18), 90.3 (C-16), 63.1 (C-17), 51.3 (C-9), 50.9 (C-15), 44.3 (C-8), 44.0 (C-13), 43.5 (C-5), 40.8 (C-7), 38.5 (C-10), 38.0 (C-14), 35.5 (C-1), 25.8 (C-12), 23.1 (C-6), 22.6 (C-24), 21.0 (C-22), 20.6 (C-2), 18.8 (C-11), 13.5 (C-20) ppm; MS (ESI, MeOH): *m*/*z* (%) = 423.1 ([M+Na]^+^, 100%); analysis: calculated for C_24_H_32_O_5_ (400.51): C 71.97, H 8.05; found: C 71.68, H 8.33.

Data for **14**: m.p. 164–165 °C (lit. [64], m.p. 173 °C); R_f_ = 0.32 (silica gel, hexanes/ethyl acetate, 7:3); ${\left[\alpha \right]}_{D}^{20}$ = −88.2° (*c* = 0.31, CHCl_3_); UV–vis (CHCl_3_): λ_max_ (log ε) = 268 nm (2.6); IR (KBr): ν = 2929 w, 2846 w, 1717 s, 1388 m, 1371 w, 1255 vs, 1136 m, 1040 s cm^−1^; ^1^H NMR (400 MHz, CDCl_3_): δ = 7.39 (*d*, *J* = 1.6 Hz, 1H, 19-H), 6.29 (*d*, *J* = 1.8 Hz, 1H, 18-H), 4.35 (*s*, 1H, 16-OH), 4.21 (*d*, *J* = 11.3 Hz, 1H, 17-H_a_), 4.05 (*d*, *J* = 11.3 Hz, 1H, 17-H_b_), 2.55 (*d*, *J* = 8.3 Hz, 2H, 2-H_a_ + 2-H_b_), 2.20 (*dd*, *J* = 12.6, 2.2 Hz, 1H, 5-H), 2.02 (*s*, 3H, 22-H), 1.99 (*m*, 2H, 1-H_b_+ 13-H), 1.89 (*d*, *J* = 11.4 Hz, 1H, 14-H_a_), 1.81–1.74 (*m*, 1H, 6-H_a_), 1.68–1.55 (*m*, 6H, 11-H_a_ + 11-H_b_ + 14-H_b_ + 7-H_a_ + 7-H_b_ + 15-H_a_), 1.55–1.35 (*m*, 4H, 12-H_a_ + 12-H_b_ + 6-H_b_ + 15-H_b_), 1.24–1.12 (*m*, 2H, 1-H_b_ + 9-H), 0.76 (*s*, 3H, 20-H) ppm; ^13^C NMR (100 MHz, CDCl_3_): δ = 171.1 (C-21), 148.5 (C-3), 141.4 (C-19), 120.3 (C-4), 109.0 (C-18), 78.7 (C-16), 68.4 (C-17), 53.6 (C-15), 52.0 (C-9), 45.5 (C-13), 44.6 (C-8), 44.1 (C-5), 40.9 (C-7), 38.6 (C-10), 38.2 (C-14), 35.5 (C-1), 26.2 (C-12), 23.2 (C-6), 21.3 (C-22), 20.6 (C-2), 18.9 (C-11), 13.6 (C-20) ppm; MS (ESI, MeOH): *m*/*z* (%) = 381.1 ([M+Na]^+^, 100%); analysis: calculated for C_22_H_30_O_4_ (358.48): C 73.71, H 8.44; found: C 73.51, H 8.69.

### 4.12. 16,17-Diacetoxy-3-oxo-4,4-dinorkauran-4-ylidene Acetic Acid (***15***)

Oxidation of **13** (65 mg, 0.17 mmol) with BAIB (119 mg, 0.37 mmol) and TEMPO (12 mg, 0.08 mmol) in acetonitrile/water (1:0.5, 20 mL) as described above, followed by chromatography (silica gel, hexanes/ethyl acetate, 6:4), gave **15** (70 mg, 99%) as a colorless solid; m.p. 207–208 °C; R_f_ = 0.25 (silica gel, hexanes/ethyl acetate, 6:4); ${\left[\alpha \right]}_{D}^{20}$ = −203.9° (*c* = 0.37, CHCl_3_); UV–vis (CHCl_3_): λ_max_ (log ε) = 265 nm (2.7); IR (ATR): ν = 3488 m, 2936 m, 1762 vs, 1746 vs, 1724 s, 1658 m, 1458 m, 1382 m, 1370 m, 1252 s, 1218 s, 1164 m, 1106 m, 1040 s cm^−1^; ^1^H NMR (400 MHz, DMSO-d_6_): δ = 7.23 (*s*, 1H, 19-OH), 5.73 (*d*, *J* = 1.3 Hz, 1H, 18-H), 4.90 (*d*, *J* = 12.4 Hz, 1H, 17-H_a_), 4.31 (*d*, *J* = 12.4 Hz, 1H, 17-H_b_), 2.38 (*d*, *J* = 7.7 Hz, 1H, 5-H), 2.22 (*d*, *J* = 9.9 Hz, 1H, 13-H), 2.17–2.08 (*m*, 1H, 2-H_a_), 2.01 (*s*, 3H, 22-H), 1.97–1.89 (*m*, 1H, 14-H_a_), 1.92 (*s*, 3H, 24-H), 1.85 (*d*, *J* = 3.0 Hz, 2H, 15-H_a_ + 15-H_b_), 1.82–1.64 (*m*, 2H, 1-H_a_ + 2-H_b_), 1.62–1.42 (*m*, 8H, 11-H_a_+ 11-H_b_ + 12-H_a_ + 12-H_b_ + 6-H_a_ + 6-H_b_ + 7-H_a_ + 7-H_b_), 1.46–1.38 (*m*, 1H, 14-H_b_), 1.32 (*d*, *J* = 6.3 Hz, 1H, 9-H), 1.23–1.11 (*m*, 1H, 1-H_b_), 0.78 (s, 3H, 20-H) ppm; ^13^C NMR (100 MHz, DMSO-d_6_): δ = 172.8 (C-3), 171.0 (C-19), 170.7 (C-21), 170.5 (C-23), 112.4 (C-18), 104.9 (C-4), 90.2 (C-16), 63.1 (C-17), 52.5 (C-9), 50.7 (C-15), 46.7 C-13), 44.1 (C-8), 43.4 (C-5), 39.6 (C-7), 39.4 (C-10), 37.5 (C-14), 35.5 (C-1), 34.2 (C-2), 25.6 (C-6), 22.6 (C-24), 21.7 (C-12), 21.0 (C-22), 18.9 (C-11), 14.4 (C-20) ppm; MS (ESI, MeOH): *m*/*z* (%) = 455.1 ([M+Na]^+^, 100%); analysis: calculated for C_24_H_32_O_7_ (432.51): C 66.65, H 7.46; found: C 66.37, H 7.71.

### 4.13. 17-O-Cinnamoyl-cafestol (***16***) and 16,17-Di-O-cinnamoyl-cafestol (***17***)

Reaction of cinnamic acid chloride with **1** as described above for 1 h under reflux and overnight at 20 °C, followed by chromatography (silica gel, hexanes/ethyl acetate, 10:1), gave **16** (34%) and **17** (60%), each as a pale yellowish solid.

Data for **16**: m.p. 147–149 °C; R_f_ = 0.14 (silica gel, hexanes/ethyl acetate, 10:1); ${\left[\alpha \right]}_{D}^{20}$ = −70.6° (*c* = 0.39, CHCl_3_); IR (KBr): ν = 2592 w, 3474 br, 3023 w, 2922 m, 2852 m, 1689 s, 1633 m, 1275 s, 1203 s cm^−1^; ^1^H NMR (400 MHz, DMSO-d_6_): δ = 7.75–7.64 (*m*, 3H, 2-H, 6-H, 3′-H), 7.47–7.31 (*m*, 4H, 3-H, 4-H, 5-H, 19-H), 6.60 (*d*, *J* = 16.1 Hz, 1H, 2′-H), 6.27 (*d*, *J* = 1.9 Hz, 1H, 18-H), 4.44 (s, 1H, 16-OH), 4.32 (*d*, *J* = 11.3 Hz, 1H, 17-H_a_), 4.21 (*d*, *J* = 11.3 Hz, 1H, 17-H_b_), 2.54 (*dt*, *J* = 8.2, 2.7 Hz, 2H, 2-H_2_), 2.23–2.10 (*m*, 1H, 13-H), 2.08–1.90 (*m*, 3H, 1-Ha, 5-H, 14-H_a_), 1.85–1.71 (*m*, 1H, 6-Ha), 1.69–1.32 (*m*, 10H, 6-Hb, 7-H2, 11-H2, 12-H2, 14-Hb, 15-H_2_), 1.17 (*m*, 1H, 1-H_b_), 1.14 (*m*, 1H, 9-H), 0.75 (s, 3H, 20-H_3_) ppm; ^13^C NMR (100 MHz, DMSO-d_6_): δ = 166.9 (C-1′), 148.5 (C-3), 144.9 (C-3′), 141.3 (C-19), 134.6 (C-1), 130.8 (C-5), 129.4 (C-3), 128.7 (C-2, C-6), 120.3 (C-4), 118.7 (C-2′), 109.0 (C-18), 78.9 (C-16), 68.7 (C-17), 53.6 (C-15), 52.0 (C-9), 45.5 (C-5), 44.6 (C-8), 44.1 (C-13), 41.0 (C-7), 38.6 (C-10), 38.2 (C-14), 35.5 (C-1), 26.3 (C-12), 23.2 (C-6), 20.6 (C-2), 18.9 (C-11), 13.6 (C-20) ppm; MS (ESI, MeOH): *m*/*z* (%) = 469.2 ([M+Na]^+^, 100%); analysis: calculated for C_29_H_34_O_4_ (446.58): C 78.00, H 7.67; found: C 77.75, H 7.90.

Data for **17**: m.p. 135–138 °C; R_f_ = 0.20 (silica gel, hexanes/ethyl acetate, 10:1); ${\left[\alpha \right]}_{D}^{20}$ = −52.1° (*c* = 0.30, CHCl_3_); IR (KBr): ν = 3025 w, 2926 w, 2849 w, 1701 s, 1639 m, 1283 s cm^−1^; ^1^H NMR (400 MHz, CDCl3): δ = 7.67 and 7.64 (2 × *d*, *J* = 16.0 Hz, 2H, 3a-H, 3′b-H), 7.58–7.43 (*m*, 4H, 2a-H, 2b-H, 6a-H, 6b-H), 7.36 (*m*, 6H, 3a-H, 3b-H, 4a-H, 4b-H, 5a-H, 5b-H), 7.24 (*d*, *J* =1.8 Hz, 1H, 19-H), 6.45, 6.44 (2 × *d*, *J* = 16.0 Hz, 2H, 2′a-H,2′b-H), 6.21 (*d*, *J* = 1.8 Hz, 1H, 18-H), 5.19 (*d*, *J* = 12.3 Hz, 1H, 17-H_a_), 4.74 (*d*, *J* = 12.3 Hz, 1H, 17-H_b_), 2.70 (*d*, *J* = 3.3 Hz, 1H, 13-H), 2.63 (*m*, 2H, 2-H_2_), 2.29 (*m*, 1H, 5-H), 2.16 (*d*, *J* = 15.6 Hz, 1H, 15-H_a_), 2.13 (*m*, 1H, 14-H_a_), 2.07 (*m*, 1H, 1-H_a_), 1.93 (*d*, *J* = 15.6 Hz, 1H, 15-H_b_), 1.88–1.45 (*m*, 9H, 6-H_2_, 7-H_2_, 11-H_2_, 12-H_2_, 14-H_b_), 1.34–1.16 (*m*, 2H, 1-H_b_, 9-H), 0.86 (*s*, 3H, 20-H_3_) ppm; ^13^C NMR (100 MHz, CDCl_3_): δ = 166.7 (C-1′a), 166.4 (C-1 b), 148.7 (C-3), 145.0 (C-3′a), 144.2 (C-3′b), 140.6 (C-19), 134.5 (C-1a), 134.3 (C-1b), 130.3 (C-4a), 130.1 (C-4b), 128.81 (C-3a, C-5a), 128.80 (C-3b, C-5b), 128.09 (C-2a, C-6a), 128.06 (C-2b, C-6b), 120.0 (C-5), 119.6 (C-2′a), 117.8 (C-2b), 108.3 (C-18), 90.8 (C-16), 63.5 (C-17), 51.8 (C-9), 51.7 (C-15), 44.5 (C-8), 44.2 (C-5), 43.6 (C-13), 40.8 (C-7), 38.7 (C-10), 38.2 (C-14), 35.7 (C-1), 25.8 (C-12), 23.1 (C-6), 20.6 (C-2), 19.1 (C-11), 13.3 (C-20) ppm; MS (ESI, MeOH): *m*/*z* (%) = 599.1 ([M+Na]^+^, 100%); analysis: calculated for C_38_H_40_O_5_ (576.72): C 79.14, H 6.99; found: C 78.81, H 7.23.

### 4.14. 17-O-(3,4,5-Trimethoxy-cinnamoyl)-cafestol (***18***)

Reaction of 3,4,5-trimethoxy cinnamoyl chloride with **1** gave **18** (75%) as a slightly yellowish solid: m.p. 208–210 °C (dec.); R_f_ = 0.30 (silica gel, hexanes/ethyl acetate, 9:1); ${\left[\alpha \right]}_{D}^{20}$ = −63.3° (*c* = 0.33, CHCl_3_); IR (KBr): ν = 3500 br, 3023 w, 2931 m, 2846 m, 1706 m, 1634 m, 1124 s; UV–vis (CHCl_3_): λ_max_ (log ε) = 242 nm (4.01), 339 nm (3.92); ^1^H NMR (400 MHz, DMSO-d_6_): δ = 7.63 (*d*, *J* = 15.9 Hz, 1H, 3′-H), 7.37 (*d*, *J* = 1.9 Hz, 1H, 19-H), 7.03 (*s*, 2H, 2-H, 6H), 6.62 (*d*, *J* = 15.9 Hz, 1H, 2′-H), 6.27 (*d*, *J* = 1.9 Hz, 1H, 18-H), 4.41 (*s*, 1H, 16-OH), 4.31 (*d*, *J* = 11.4 Hz, 1H, 17-H_a_), 4.21 (*d*, *J* = 11.4 Hz, 1H, 17-H_b_), 3.86 (*s*, 6H, 3-OMe, 5-OMe), 3.68 (s, 3H, 4-OMe), 2.54 (*m*, 2H, 2-H_2_), 2.19 (*m*, 1H, 13-H), 2.09–2.00 (*m*, 2H, 1-H_a_, 5-H), 1.90 (*d*, *J* = 11.4 Hz, 1H, 14-H_a_), 1.77–1.36 (*m*, 11H, 6-H2, 7-H2, 11-H_2_, 12-H_2_, 14-H_b_, 15-H_2_), 1.20 (*m*, 1H, 1-H), 1.15 (*m*, 1H, 9-H), 0.75 (*s*, 3H, 20-H_3_) ppm; ^13^C NMR (100 MHz, DMSO-d_6_): δ = 167.1 (C-1′), 153.5 (C-3, C-5), 148.5 (C-3), 141.3 (C-4), 145.1 (C-3′), 139.9 (C-19), 130.2 (C-1), 120.3 (C-4), 118.0 (C-2′), 109.0 (C-18), 106.3 (C-2, C-6), 78.9 (C-16), 68.6 (C-17), 60.5 (OMe-4), 56.5 (OMe-3, OMe-5), 53.7 (C-15), 52.0 (C-9), 45.1 (C-5), 44.4 (C-8), 44.1 (C-13), 41.0 (C-7), 38.6 (C-10), 38.2 (C-14), 35.5 (C-1), 26.2 (C-12), 23.2 (C-6), 20.6 (C-2), 18.9 (C-11), 13.6 (C-20) ppm; MS (ESI, MeOH): *m*/*z* (%) = 559.5 ([M+Na]^+^, 100%); analysis: calculated for C_32_H_40_O_7_ (536.67): C 71.62, H 7.51; found: C 71.45, H 7.83.

### 4.15. 17-O-(3,4-Dihydroxy-cinnamoyl)-cafestol (***19***)

Reaction of caffeic acid chloride with **1** gave **19** (73%) as a slightly yellowish solid: m.p. 112–115 °C; R_f_ = 0.14 (silica gel, CHCl_3_); ${\left[\alpha \right]}_{D}^{20}$ = −27.1° (*c* = 0.26, MeOH); IR (Kr): ν = 3475 m, 3200 r, 3010 s, 2922 m, 1671 s, 1604 s, 1276 s cm^−1^; UV–vis (MeOH): λ_max_ (log ε) = 223 nm (4.55), 360 nm (4.60); ^1^H NMR (400 MHz, CD_3_OD): δ = 9.30 (*s*, 1H, 3-OH), 9.04 (*s*, 1H-4-OH), 7.53 (*d*, *J* = 15.9 Hz, 3′H), 7.23 (*d*, *J* = 1.8 Hz, 1H, 19-H), 7.03 (*d*, *J* = 2.2 Hz, 1H, 2-H), 6.93 (*d*, *J* = 8.2 Hz, 1H, 5-H), 6.77 (*d*, *J* = 8.3 Hz, 2H, 6-H), 6.29–6.17 (*m*, 2H, 2′-H, 18-H), 4.63 (*s*, 1H, 16-OH), 3.70–3.52 (*m*, 2H, 17-H_2_), 2.56 (*m*, 2H, 2-H_2_), 2.24–1.97 (*m*, 4H, 1-H_a_, 5-H, 13-H, 14-H_a_), 1.93–1.11 (*m*, 13H, 1-H_b_, 6-H_2_, 7-H2, 9-H, 11-H_2_, 12-H_2_, 14-H_b_, 15-H_2_), 0.82 (*s*, 3H, 20-H_3_) ppm; ^13^C NMR (100 MHz, CD_3_OD): δ = 168.4 (C-1′), 148.1 (C-3), 145.5 (C-3, C-4), 145.4 (C-2′), 140.2 (C-19), 126.3 (C-1), 121.5 (C-4), 115.1 (C-6), 113.7 (C-5, C-2), 113.4 (C-3′), 107.7 (C-18), 65.5 (C-16), 63.4 (C-17), 52.7 (C-9), 52.2 (C-15), 50.6 (C-10), 44.3 (C-5), 44.2 (C-8), 44.0 (C-13), 40.7 (C-7), 37.8 (C-14), 35.5 (C-1), 25.9 (C-12), 22.8 (C-6), 20.0 (C-2), 18.6 (C-11), 12.3 (C-20) ppm; MS (ESI, MeOH): *m*/*z* (%) = 501.2 ([M+Na]^+^, 80%), 491.3 ([M-H]-, 70%); analysis: calculated for C_29_H_34_O_6_ (478.59): C 72.78, H 7.16; found: C 72.45, H 7.30.

## Figures and Tables

**Table 1:** Full NMR spectroscopic data for **1** and **2** (CDCl_3_, 600 MHz for ^1^H NMR and 151 MHz for ^13^C NMR, 27 °C; numbering scheme according to Figure 1).

	Kahweol (2)			Cafestol (1)		
Atom Number	^1^H NMR Shift in ppm	Coupling in Hz	^13^C NMR Shift in ppm	^1^H NMR Shift in ppm	Coupling in Hz	^13^C NMR Shift in ppm
1	5.88	*d*, 10.0	138.6	2.03/1.22	*m*/*dt*, 13.1, 9.3	35.9
2	6.23	*d*, 10.0	115.5	2.60	*dt*, 9.4, 3.6	20.7
3	-	-	150.3	-	-	148.8
4	-	-	121.9	-	-	120.2
5	2.58	*dd*, 12.8, 3.2	44.6	2.25	*ddt*, 12.7, 2.5, 2.5	44.4
6	1.87/1.75	*ddd*, 12.9, 6.5, 3.2/*ddd*, 12.7, 12.6, 4.0	22.2	1.80/1.51	*ddt*, 13.0, 3.2/*m*	23.2
7	1.64	* m *	40.5	1.66	* m *	50.0
8	-	-	45.2	-	-	44.8
9	1.56	*t*, 4.74	48.4	1.17	*d*, 8.0	52.3
10	-	-	42.0	-	-	38.8
11	1.70	* m *	19.1	1.70/1.62	* m *	19.1
12	1.64/1.52	* m *	26.0	1.61/1.53	* m *	26.1
13	2.05	*d*, 4.5	45.4	2.06	* m *	45.4
14	1.93/1.66	*d*, 11.6/-	38.3	2.04/1.66	*d*, 13.0	38.3
15	1.64/1.51	*d*, 14.7	53.1	1.63/1.50	*d*, 13.1	53.4
16	-	-	82.1	-	-	82.2
17	3.79/3.68	*d*, 11.1	66.4	3.79/3.68	*d*, 11.2	66.3
18	6.29	*d*, 1.8	108.3	6.19	*d*, 1.9	108.4
19	7.26	*d*, 1.8	141.3	7.23	*dt*, 1.7, 0.8	140.7
20	0.97	* s *	15.7	0.82	* s *	13.4

**Table 2:** Cytotoxicity of compounds (IC_50_ in μM (from SRB assays; each performed in triplicate with three technical replicates each; incubation time 72 h)); cell lines: malignant: MCF7 (breast adenocarcinoma) and A2780 (ovarian carcinoma); non-malignant: NIH 3T3 (murine fibroblasts). Doxorubicin (**DX**) was used as a positive standard.

Compound	MCF7	A2780	NIH 3T3
**1**	>30	>30	>30
**2**	>30	>30	>30
**3**	7.5 ± 1.0	6.9 ± 1.1	10.3 ± 1.4
**4**	1.1 ± 0.1	1.3 ± 0.1	3.2 ± 0.4
**5**	7.1 ± 0.8	6.8 ± 1.3	5.7 ± 07
**6**	6.1 ± 0.6	6.9 ± 0.6	20.7 ± 0.9
**7**	>30	>30	>30
**8**	11.3 ± 1.5	9.1 ± 0.7	7.2 ± 0.8
**16**–**19**	>30	>30	>30
**DX**	1.1 ± 0.3	0.02 ± 0.01	1.7 ± 0.3

## Data Availability

The raw data supporting the conclusions of this article will be made available by the authors upon request.

## Supplementary Materials

The following supporting information can be downloaded at: https://www.mdpi.com/article/10.3390/molecules30112291/s1, Representative 2D NMR spectra of **1** and **2**.

## Abbreviations

The following abbreviations are used in this manuscript:

MTBE	Methyl tert-butyl ether
1D NMR	One-dimensional nuclear magnetic resonance
2D NMR	Two-dimensional nuclear magnetic resonance
gHSQC	Gradient heteronuclear single quantum coherence
HSQC	Heteronuclear single quantum coherence
HMBC	Heteronuclear multiple bond correlation
INADEQUATE	Incredible natural abundance double quantum transfer experiment
ADEQUATE	Adequate double quantum spectroscopy
TOXY-NMR	Total correlation spectroscopy with X-filtering
SNR	Signal-to-noise ratio
EDC	1-ethyl-3-(3-dimethylaminopropyl)carbodiimide
HOBt	1-hydroxybenzotriazole
TEA	Triethylamine
DMAP	4-dimethylaminopyridine
TEMPO	(2,2,6,6-tetramethylpiperidin-1-yl)oxyl
BAIB	Diacetoxyiodobenzene
NCS	N-chlorosuccinimide
SRB	Sulforhodamine B
MCF7	Human breast adenocarcinoma cell line
A2780	Human ovarian carcinoma cell line
NIH 3T3	Mouse embryonic fibroblast cell line
IC50	Half-maximal inhibitory concentration
