# Supplementary material for: Towards Cytotoxic Derivatives of Cafestol
Source: Molecules. 2025 May 23;30(11):2291. doi: 10.3390/molecules30112291 (PMC12156820; doi:10.3390/molecules30112291)

Supplementary Materials

Towards Cytotoxic Derivatives of Cafestol

Niels V. Heise, Marie Kozubek, Sophie Hoenke, Senta Ludwig, Hans-Peter Deigner, Ahmed Al-Harrasi, René Csuk*

Structure and numbering scheme:

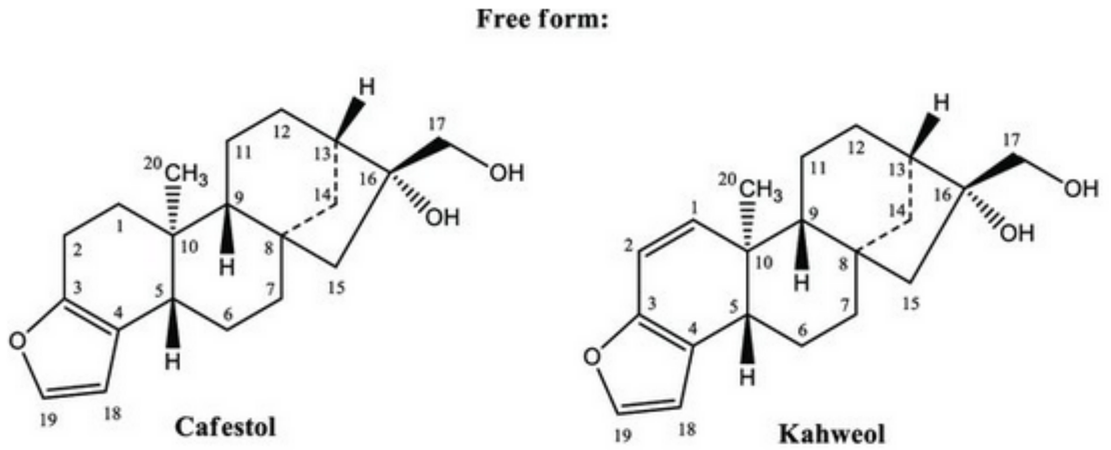

1. Cafestol:

INADEQUATE Spectrum

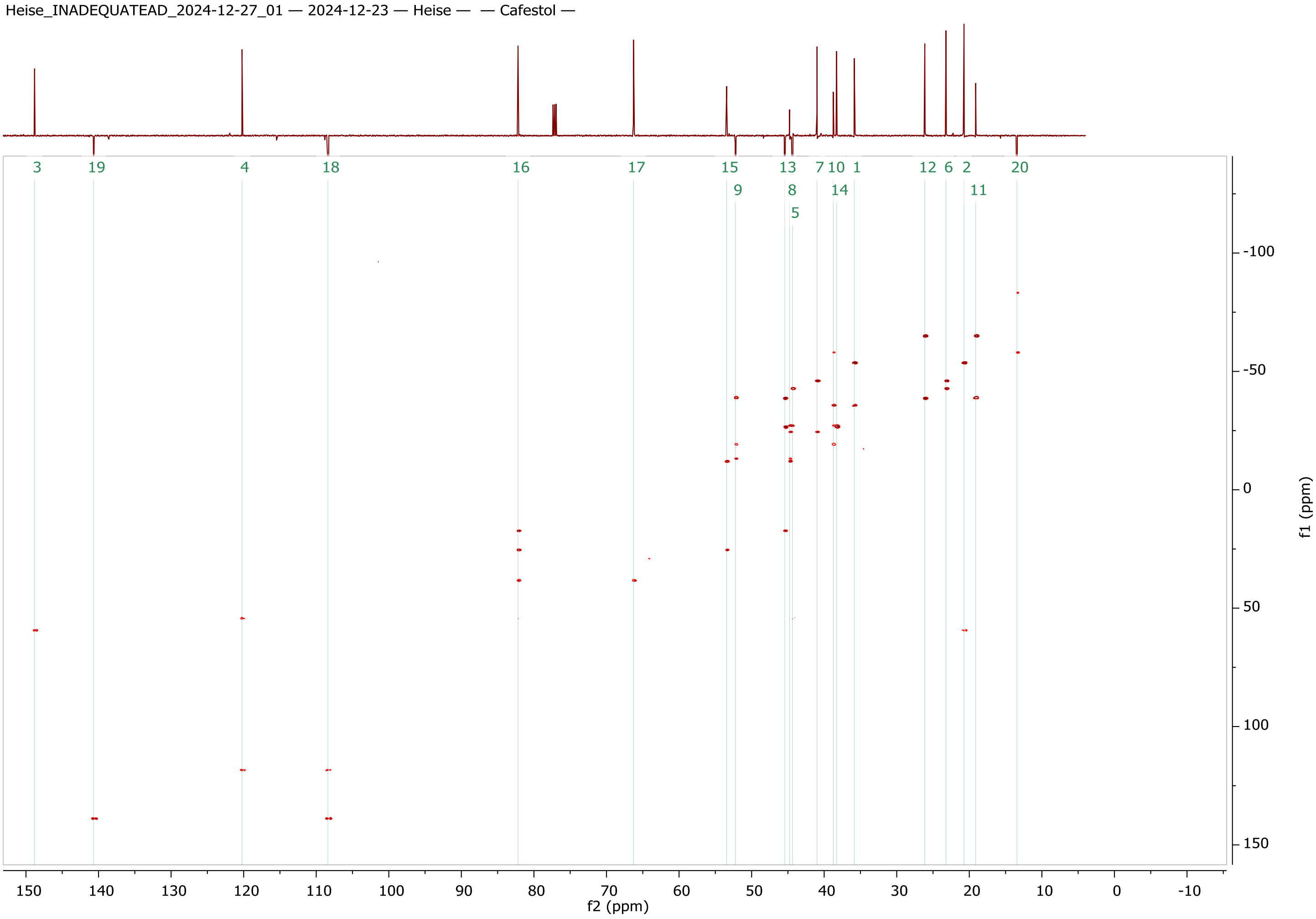

ADEQATE Spectrum

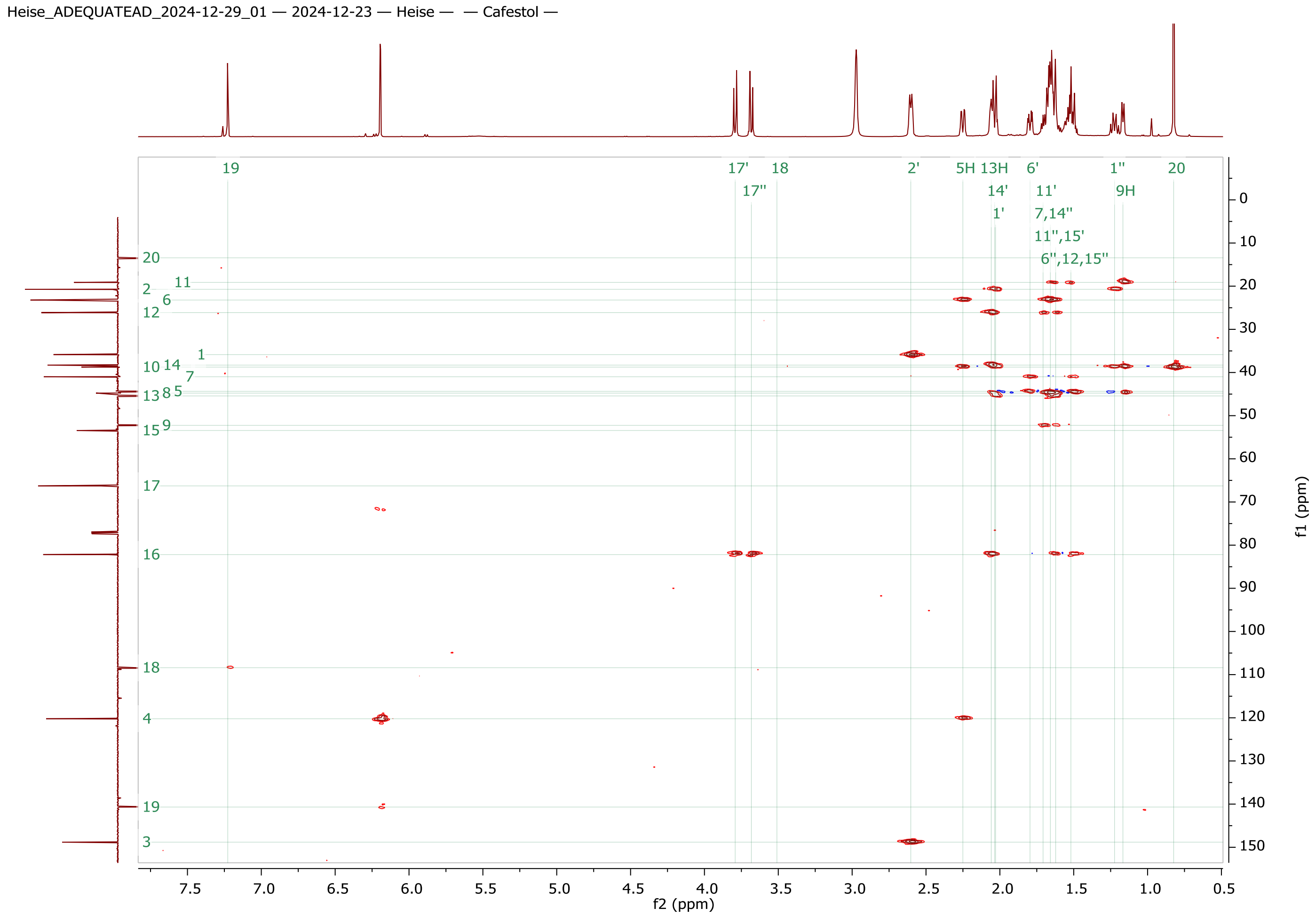

1H NMR and 13 APT NMR

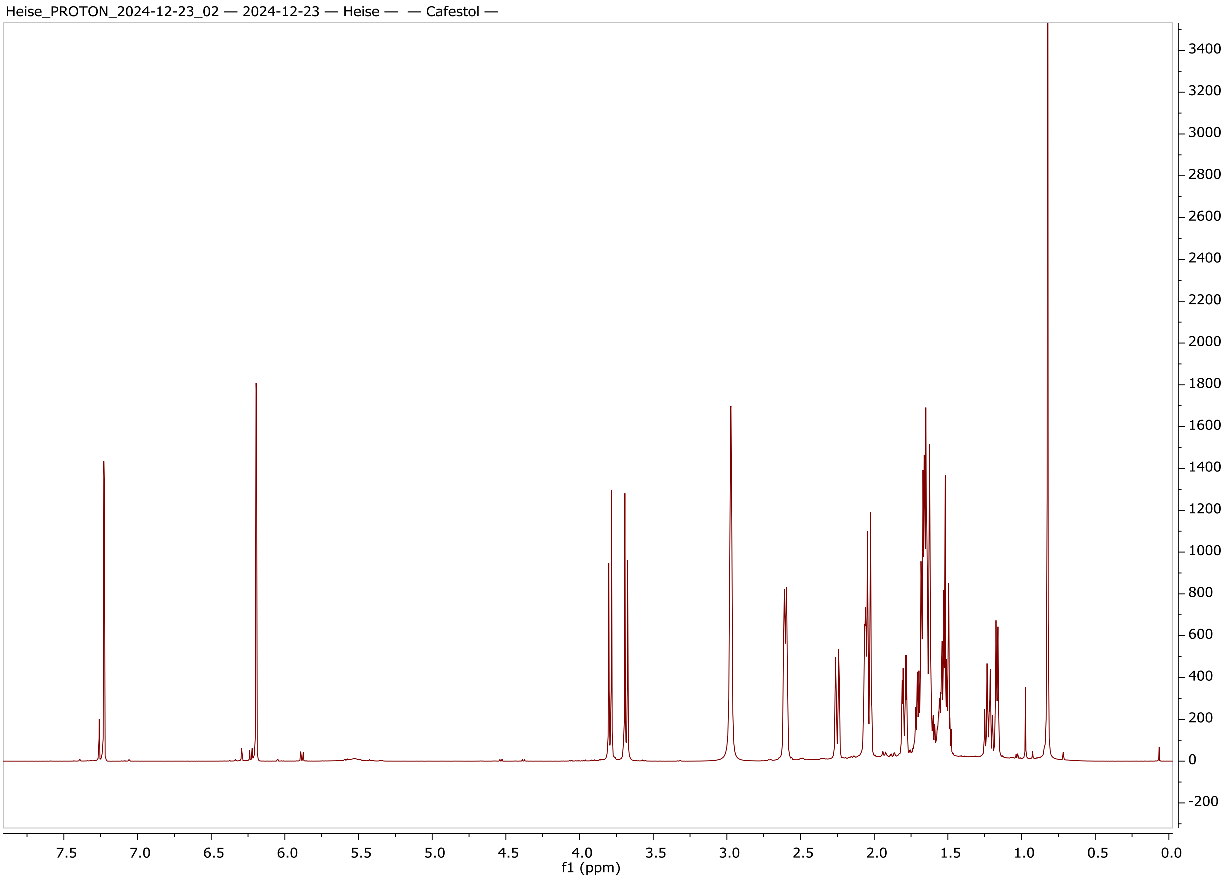

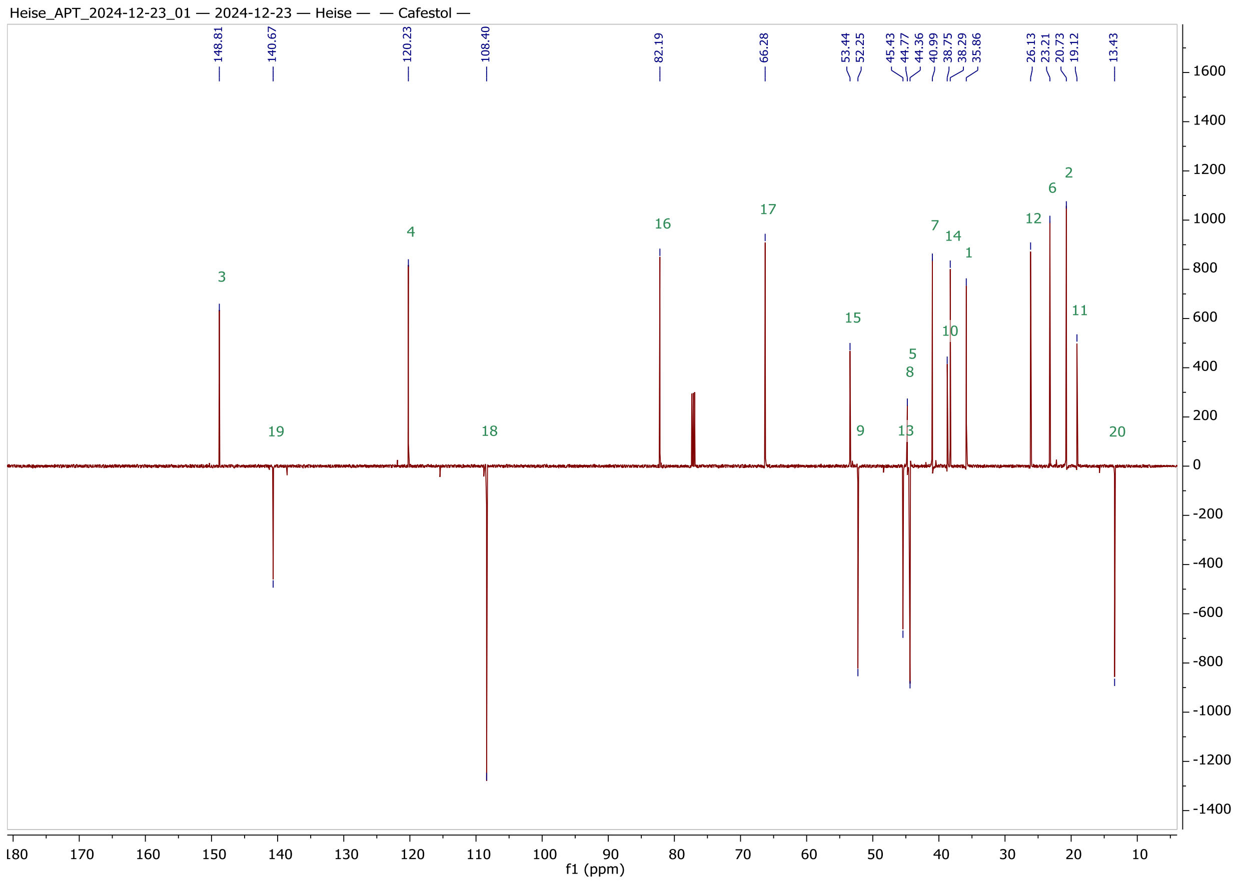

gHSQAC Spectrum

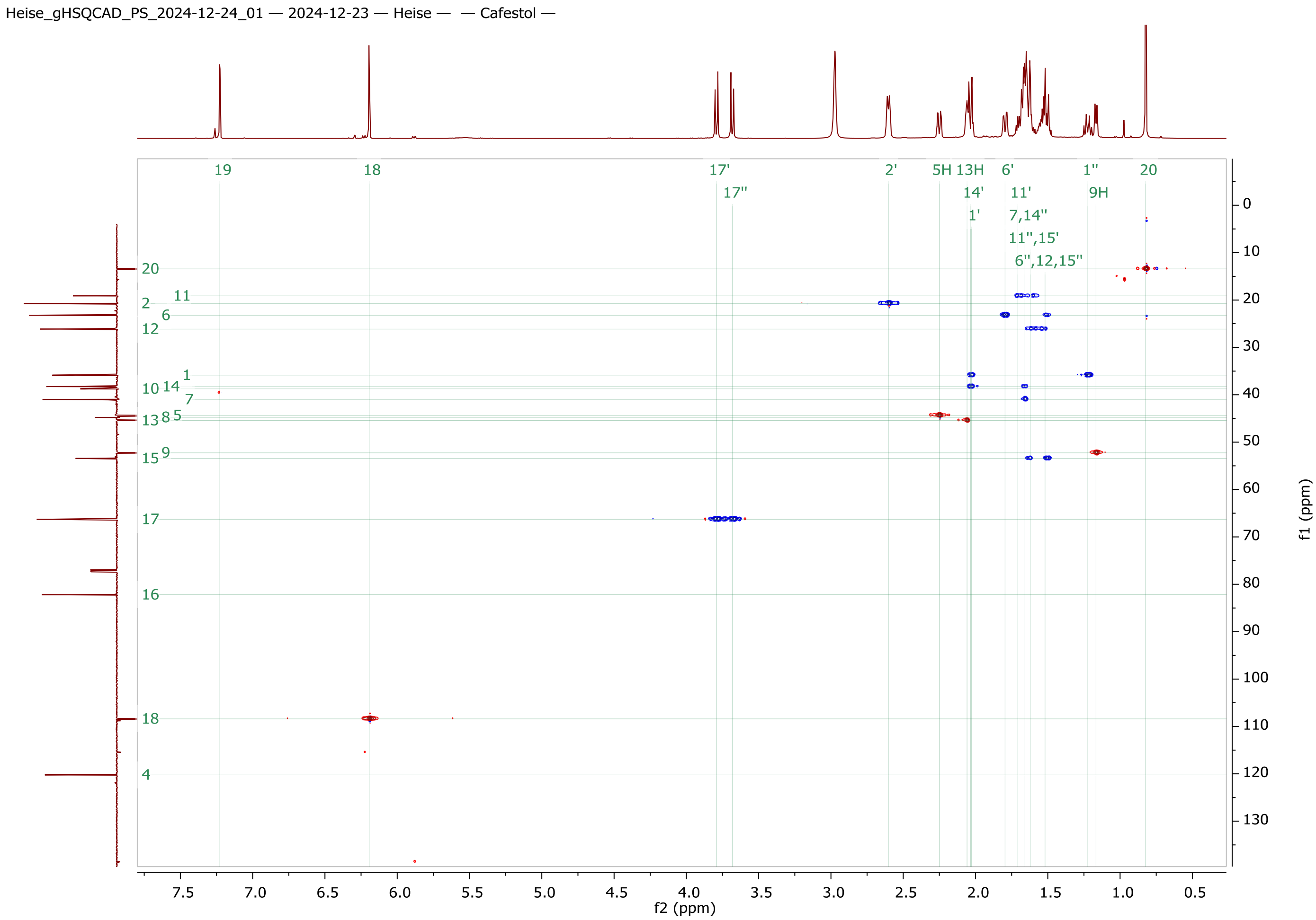

gHMBC Spectrum

Heise_gHMBCAD_2024-12-24_01 — 2024-12-23 — Heise — — Cafestol —

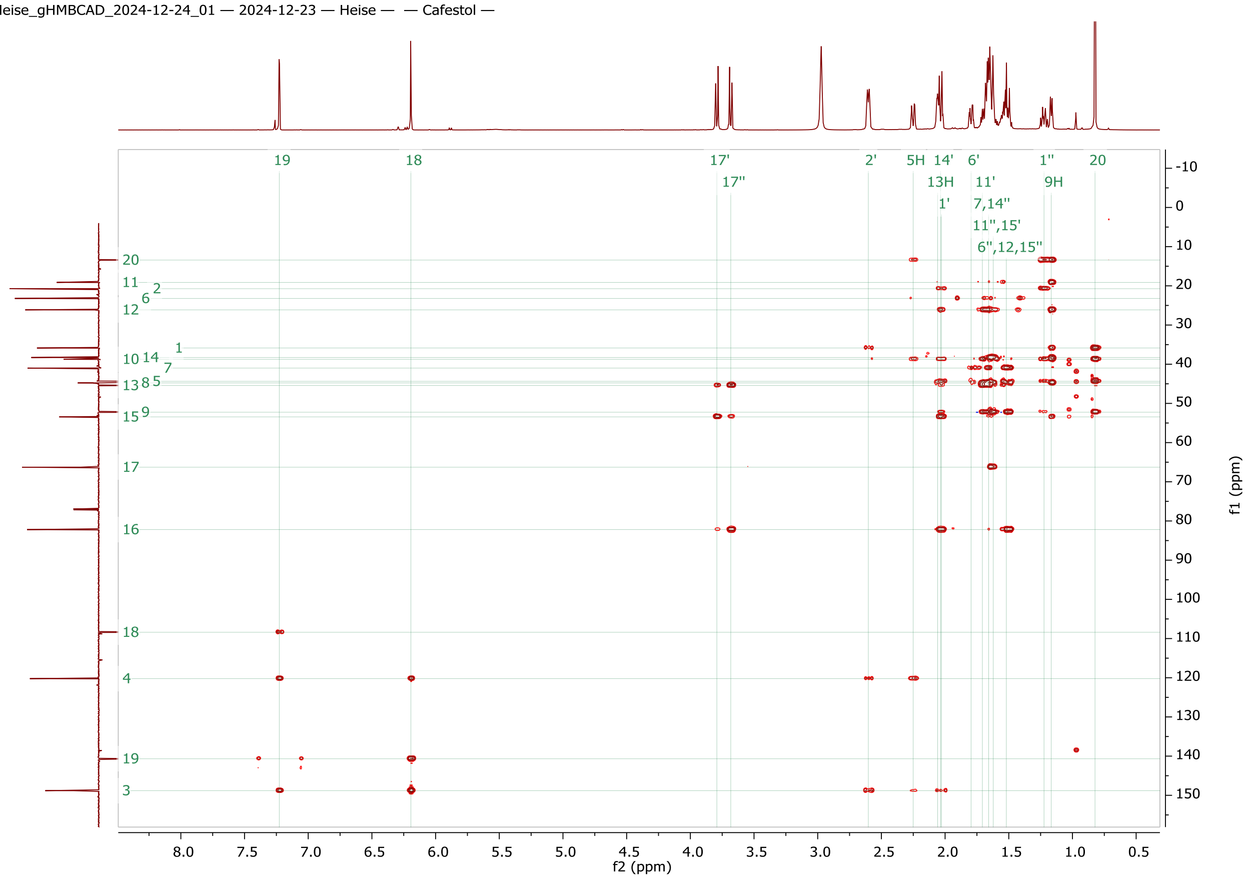

HSQC TOXY

Heise_gHSQCADTOXY_2024-12-24_01 — 2024-12-23 — Heise — — Cafestol —

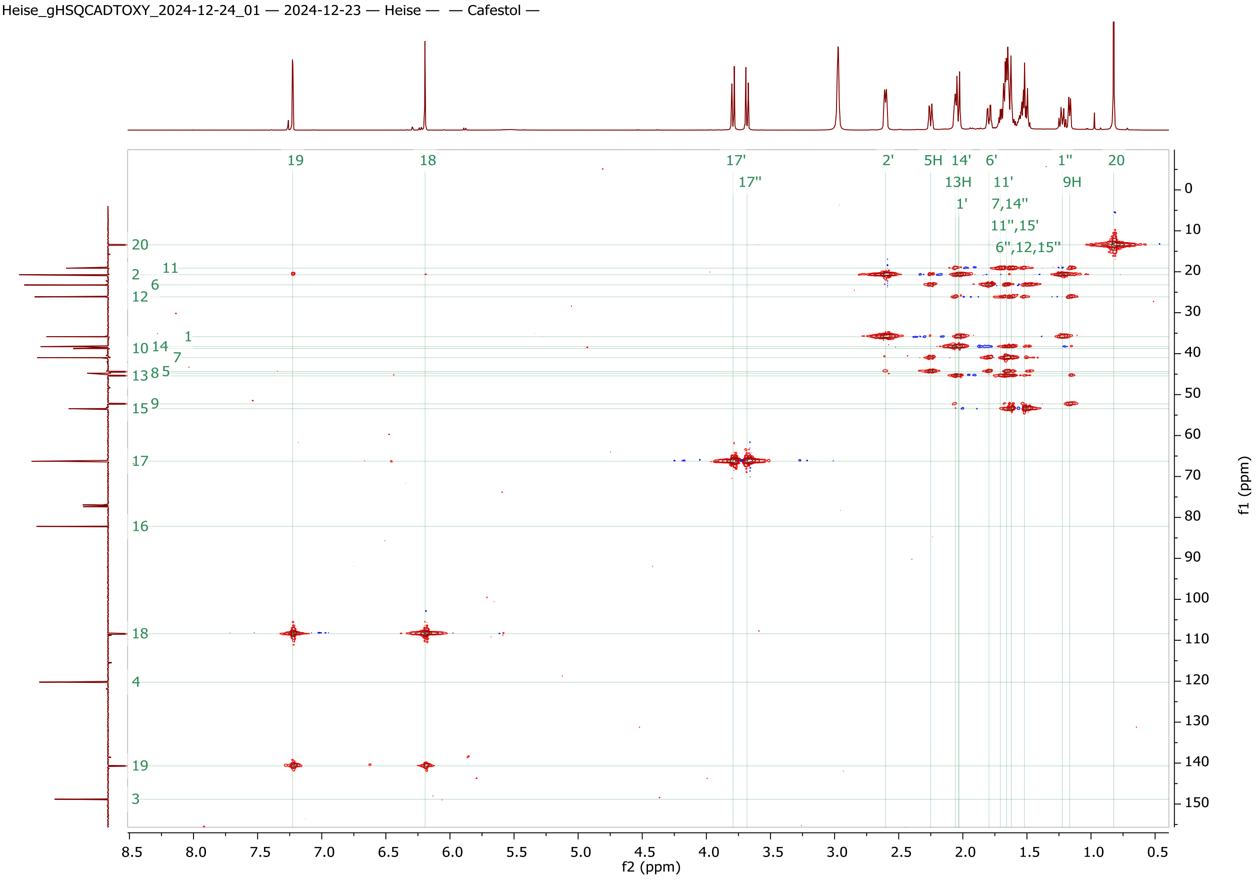

2. Kahweol

ADEQUATE NMR

Heise3_ADEQUATEAD_2025-01-06_02 — 2024-12-30 — Heise3 — Kahweol — —

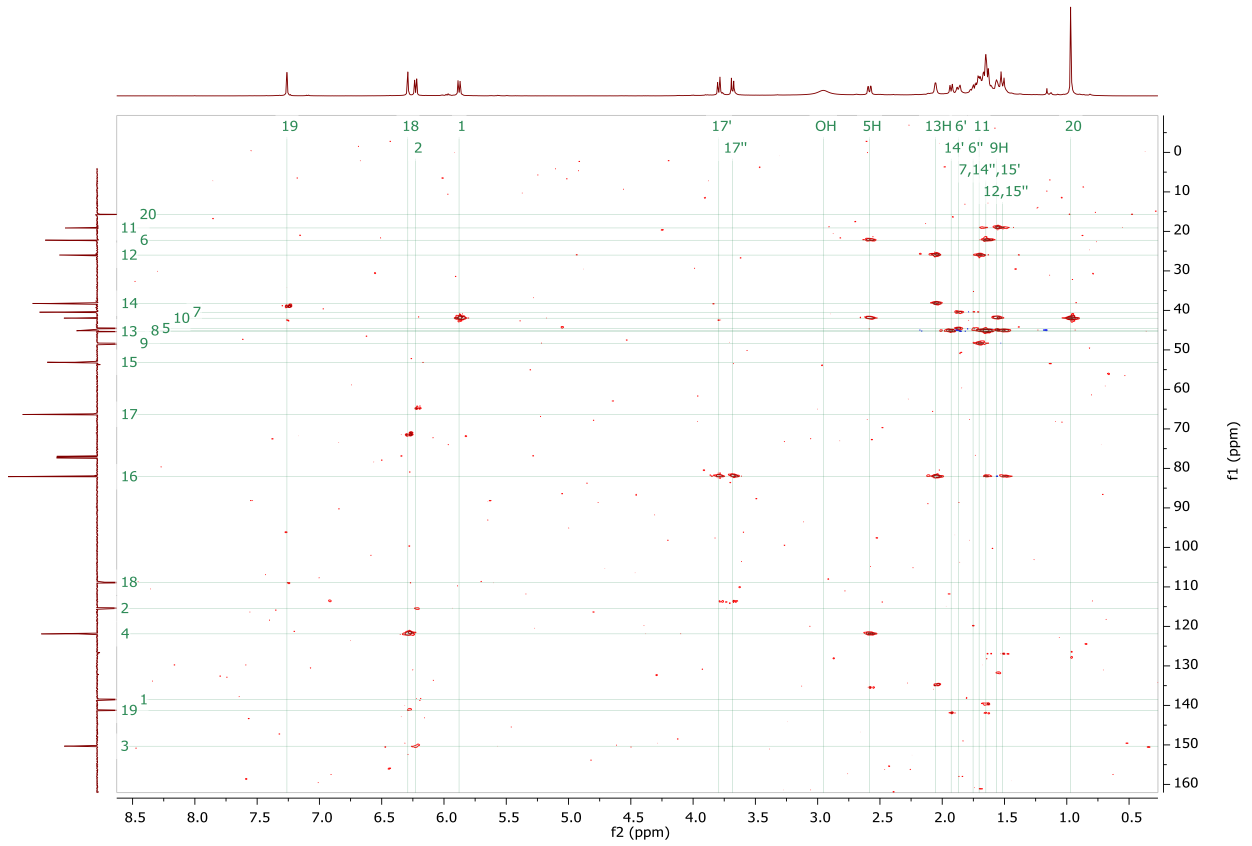

INADEQUATE NMR

Heise3_INADEQUATEAD_2025-01-04_01 — 2024-12-30 — Heise3 — Kahweol — —

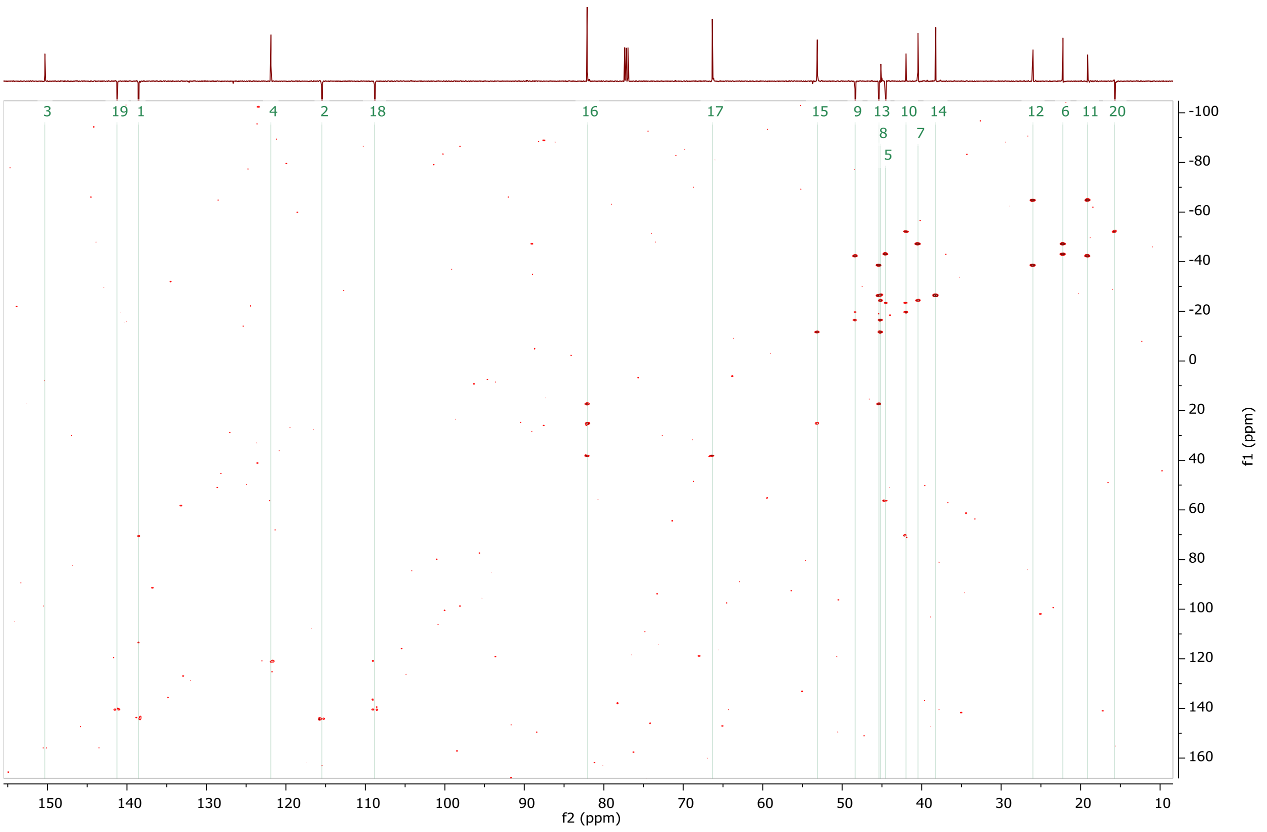

1H NMR

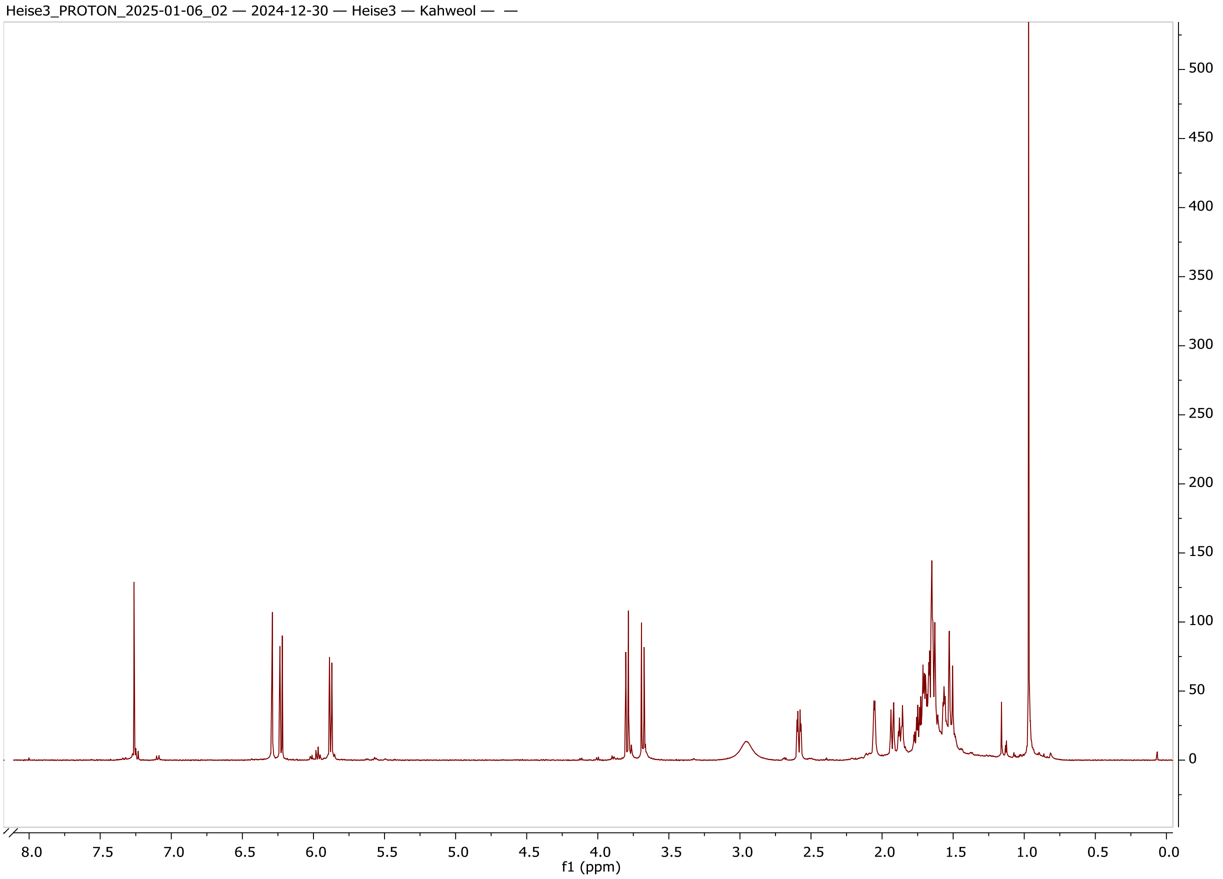

13C APT NMR

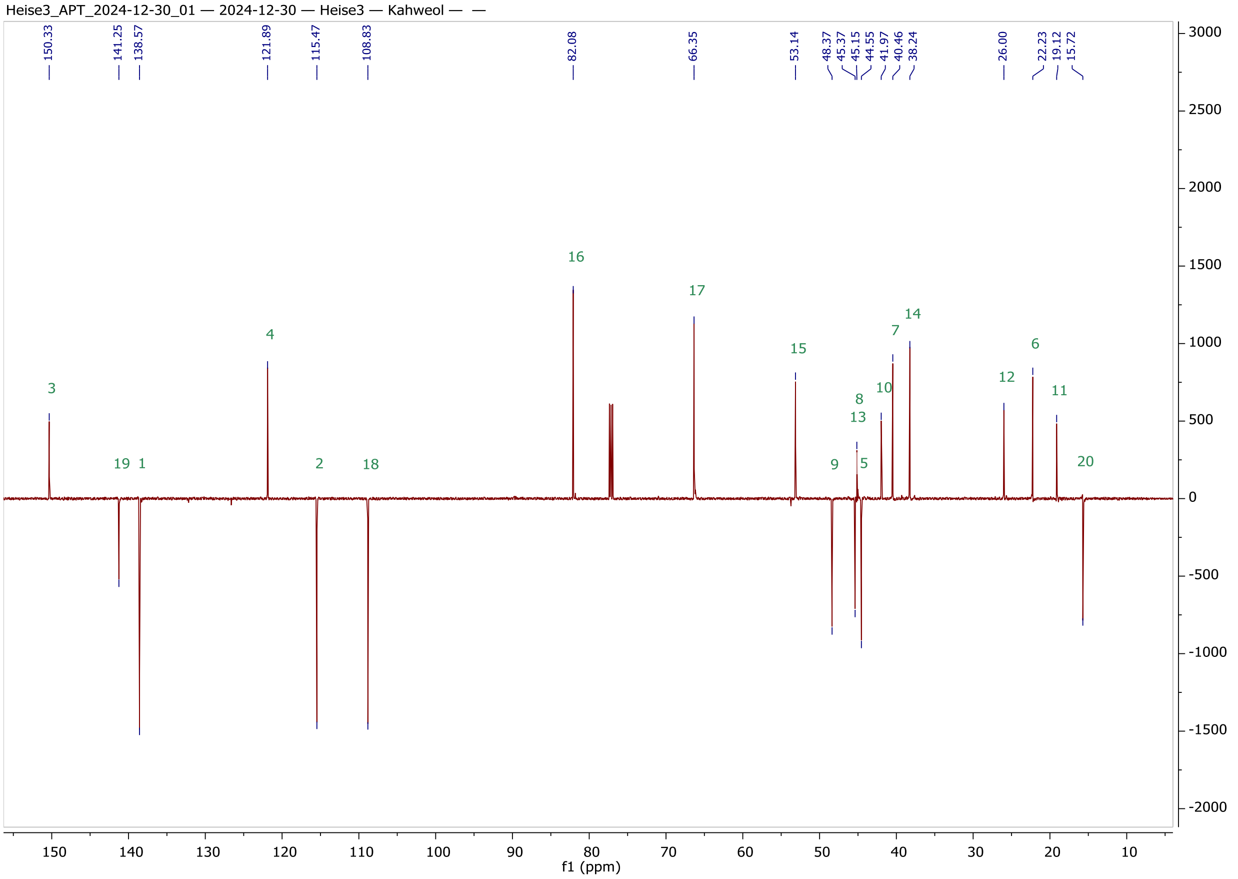

gHSQC Spectrum

Heise3_gHSQCAD_PS_2024-12-30_01 — 2024-12-30 — Heise3 — Kahweol — —

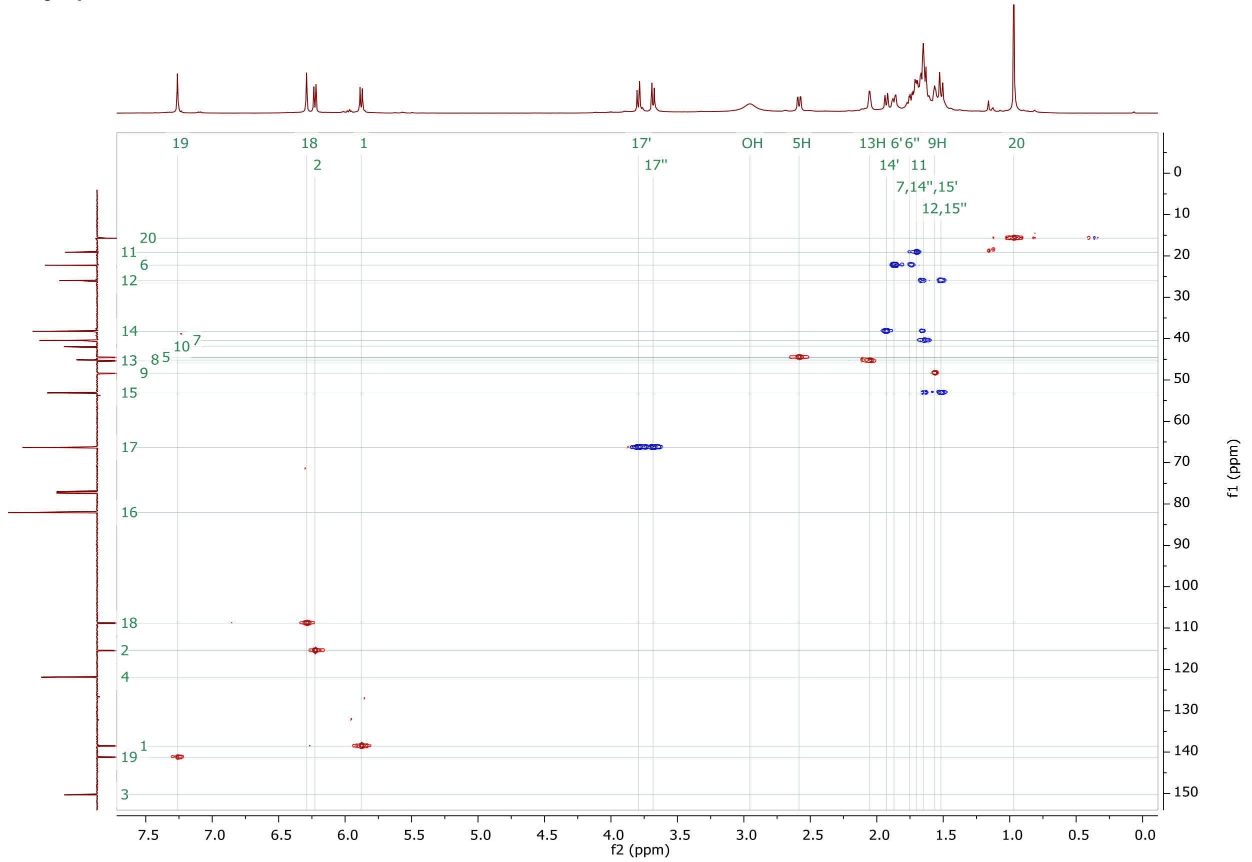

gHMBC Spectrum

Heise3_gHMBCAD_2024-12-31_01 — 2024-12-30 — Heise3 — Kahweol — —

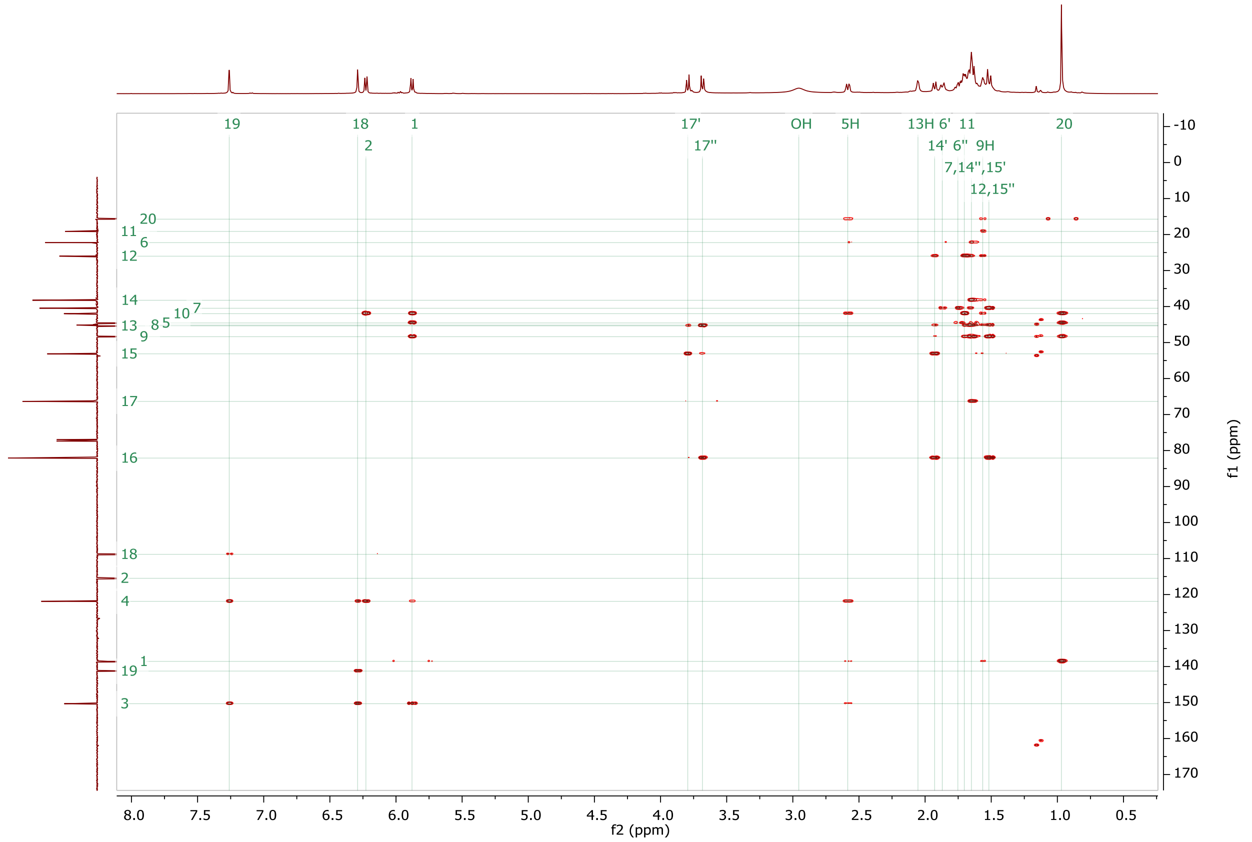

gHSQC-TOXY Spectrum

Heise3_gHSQCADTOXY_2024-12-31_01 — 2024-12-30 — Heise3 — Kahweol — —

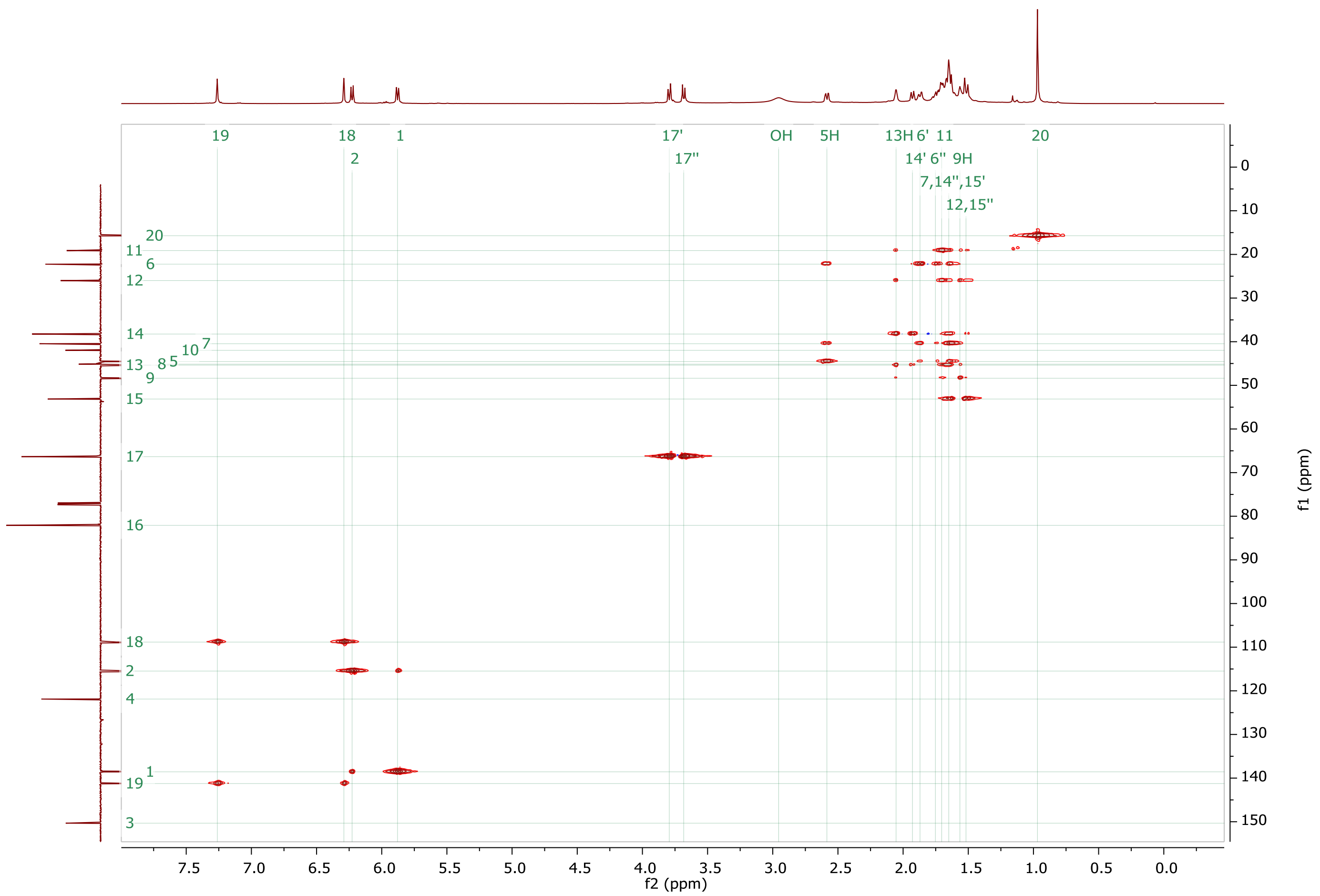

Supplement: Supplementary file 1 [file molecules-30-02291-s001.zip › molecules-3619804-supplementary.pdf]
